# Supplementary material for: Dess–Martin Periodinane-Mediated Oxidative Coupling Reaction of Isoquinoline with Benzyl Bromide
Source: Molecules. 2023 Jan 17;28(3):923. doi: 10.3390/molecules28030923 (PMC9919522; doi:10.3390/molecules28030923)
Supplement: Supplementary file 1 [file molecules-28-00923-s001.zip › molecules-2143853-supplementary.pdf]

Supplementary Materials

Dess–Martin Periodinane-Mediated Oxidative Coupling Reaction of Isoquinoline with Benzyl Bromide

Chunmei Yang [1,2,†], Guoqing Zhang [1,†], Senling Tang [1,2], Yang Pan [1,2], Huawu Shao [1] and Wei Jiao [1,*]

[1] Natural Products Research Centre, Chengdu Institute of Biology, Chinese Academy of Sciences, Chengdu 610041, China
[2] University of Chinese Academy of Sciences, Beijing 100049, China
* Correspondence: jiaowei@cib.ac.cn
† These authors contributed equally to this work.

1. ^{1}H NMR and ^{13}C NMR spectra of the synthesized compounds

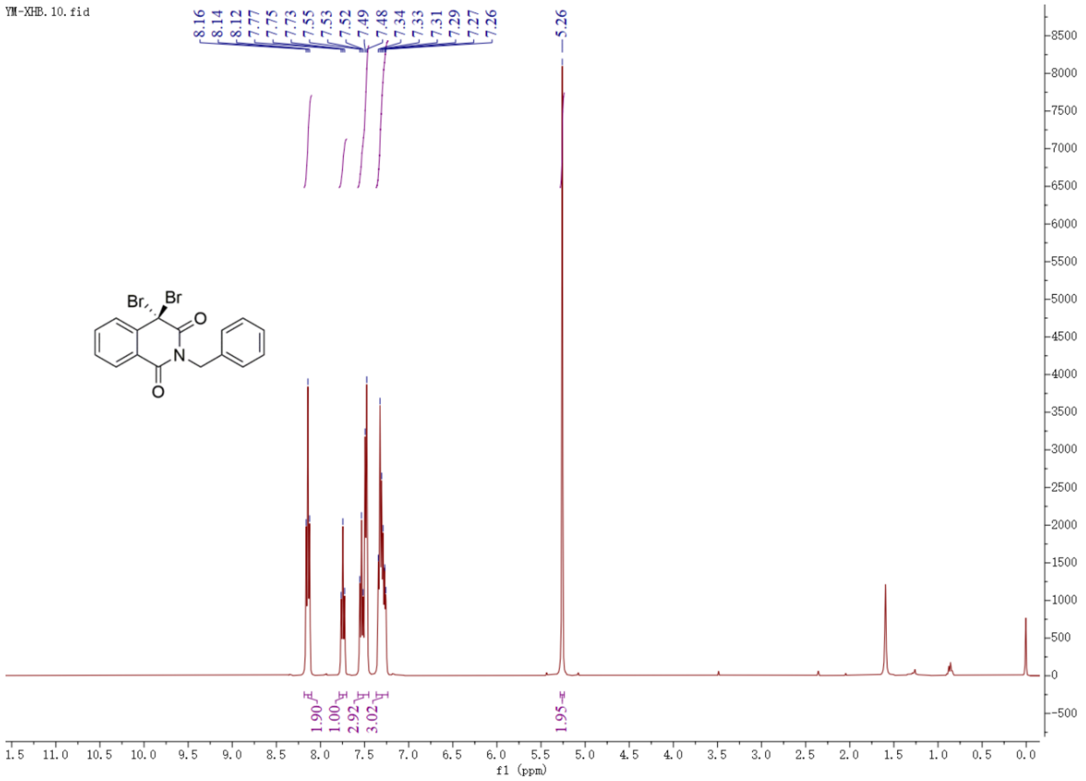

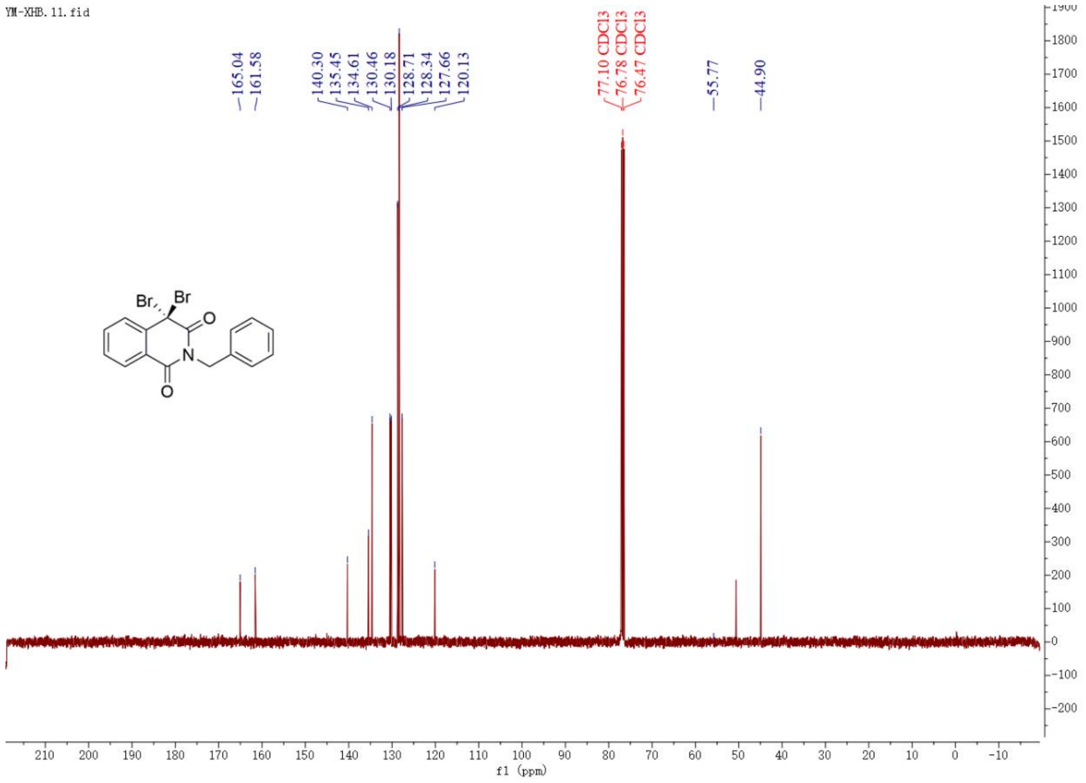

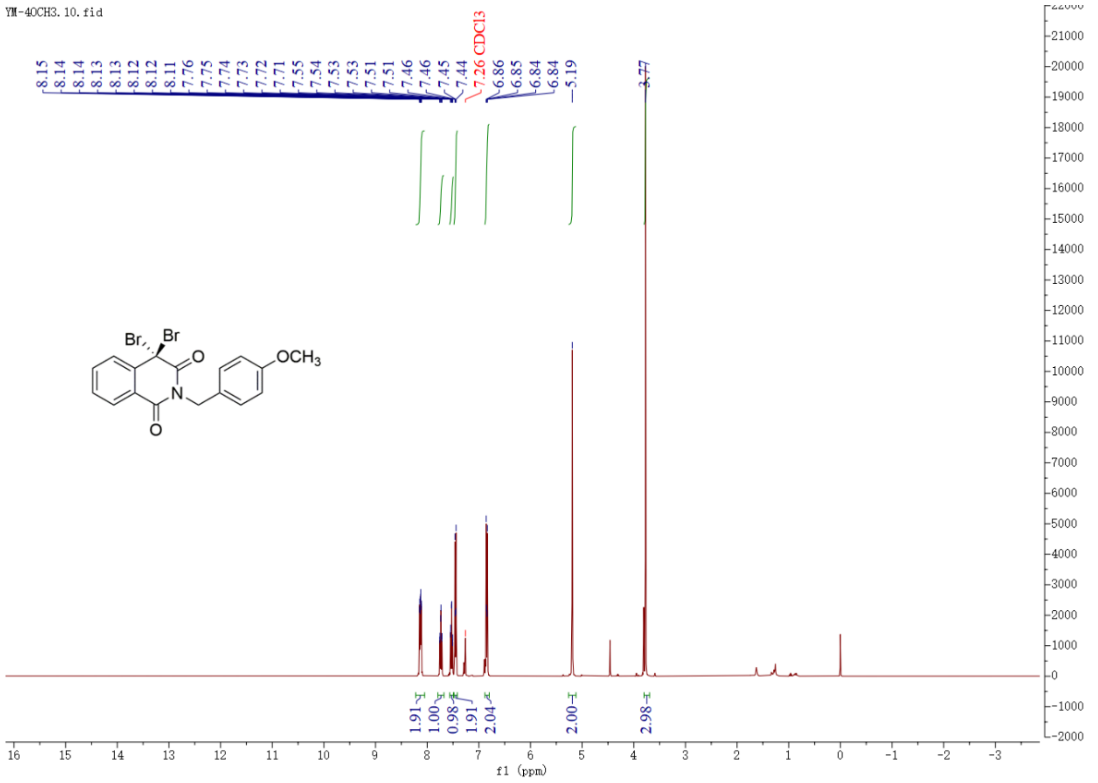

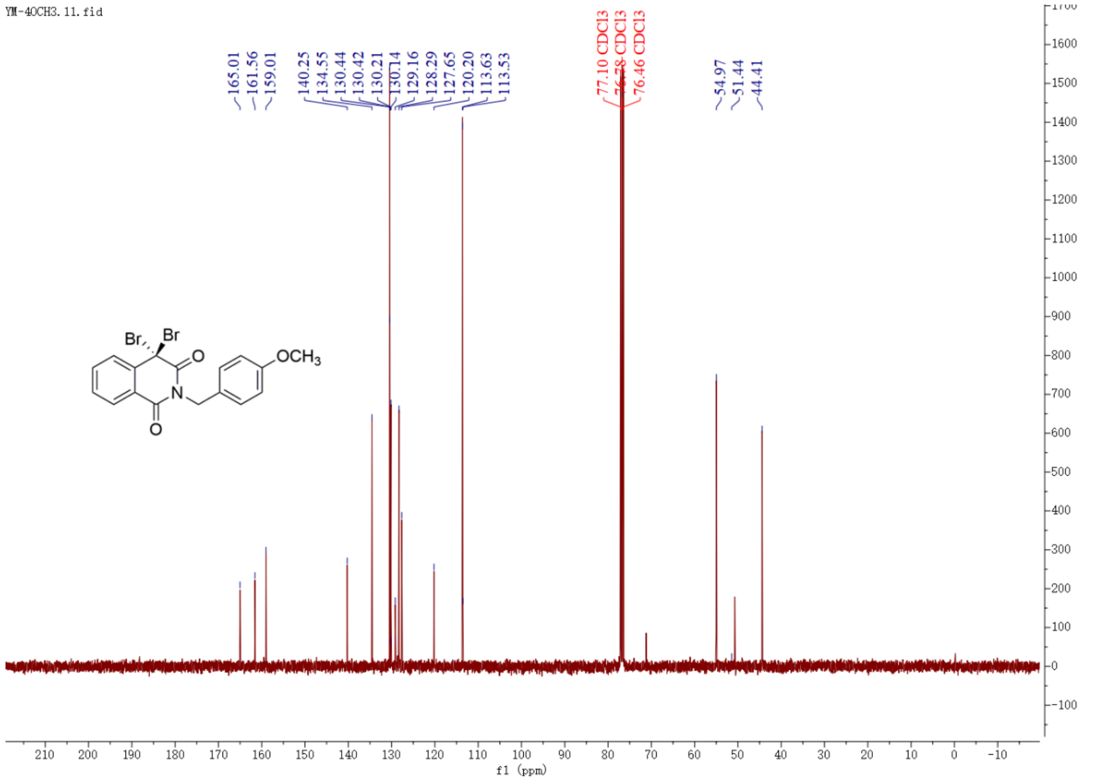

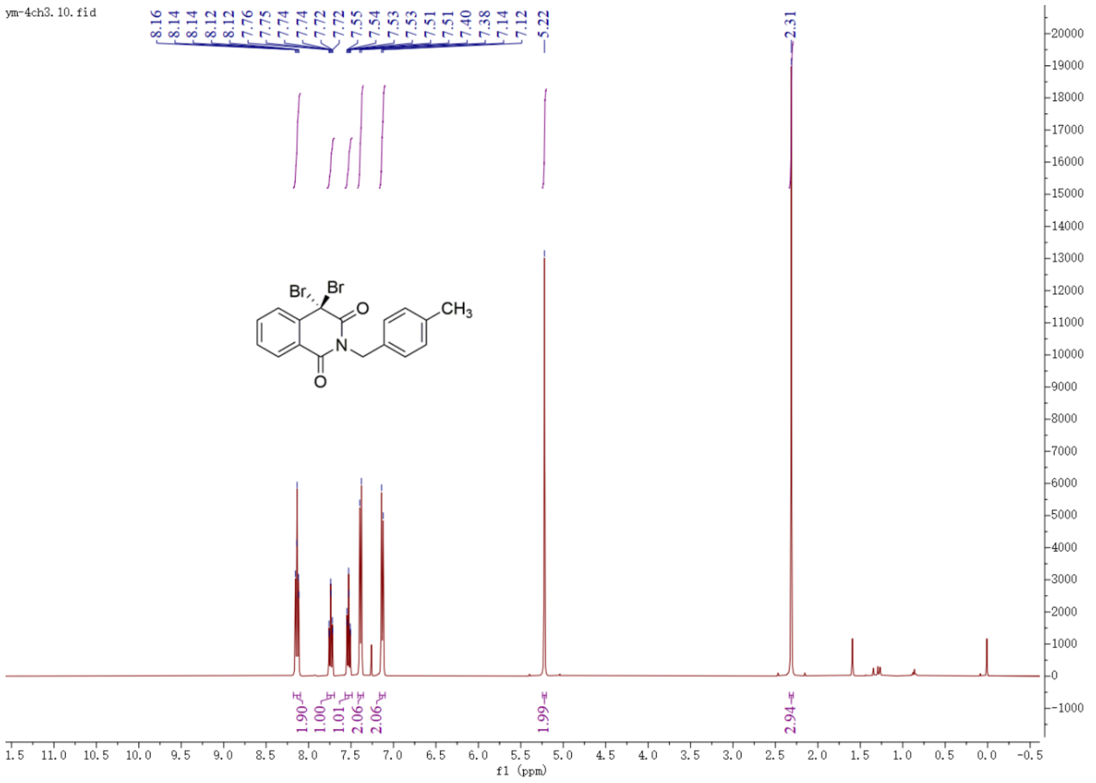
ym-4ch3.10.fid
8.16
8.14
8.14
8.12
8.12
7.76
7.75
7.74
7.74
7.72
7.72
7.55
7.54
7.53
7.53
7.51
7.51
7.40
7.38
7.14
7.12
5.22
2.31
Br
Br
O
N
O
CH3
1.90
1.00
1.01
2.06
2.06
1.99
2.94
f1 (ppm)

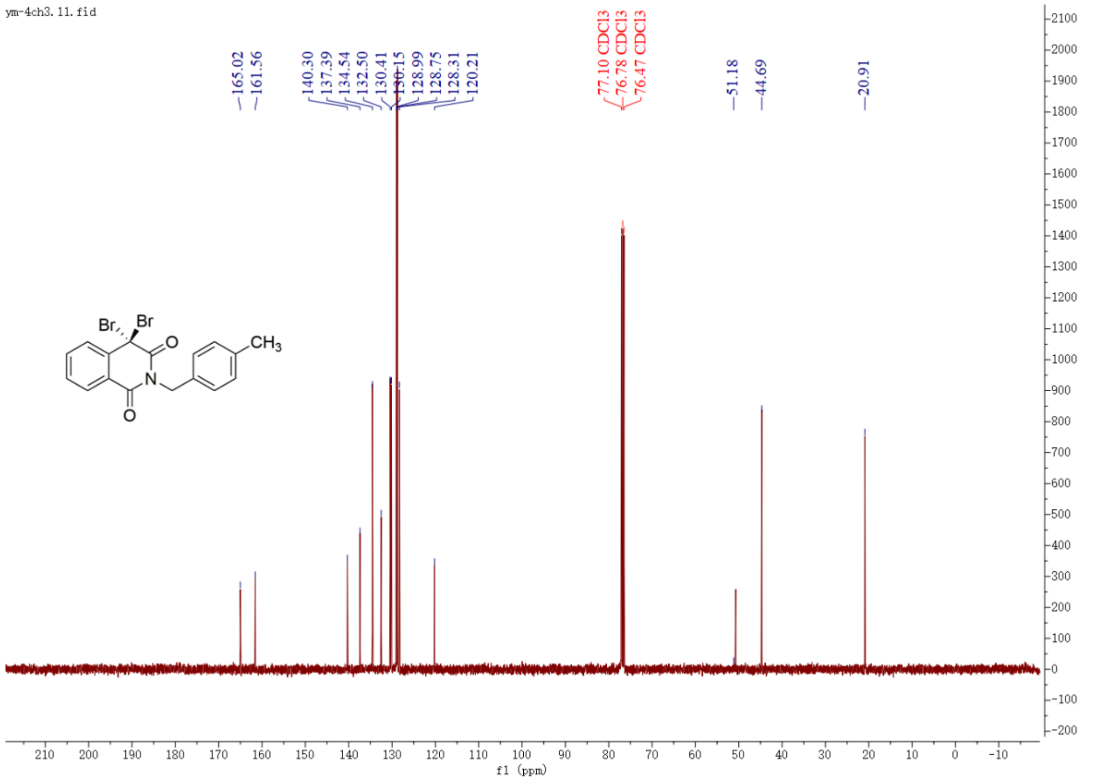
ym-4ch3.11.fid
165.02
161.56
140.30
137.39
134.54
132.50
130.41
130.15
128.99
128.75
128.31
120.21
77.10 CDCl3
76.78 CDCl3
76.47 CDCl3
51.18
44.69
20.91
Br
Br
O
N
O
CH3
f1 (ppm)

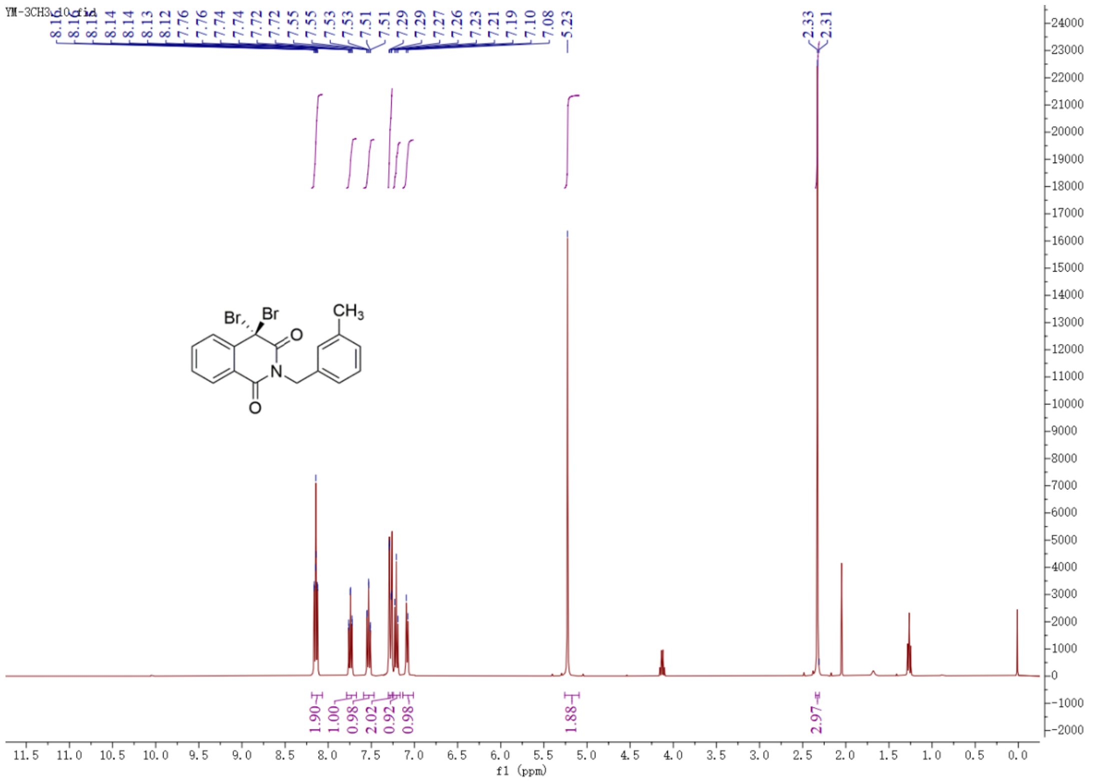
YM-3CH3.10.fid
8.16
8.16
8.15
8.14
8.14
8.13
8.12
7.76
7.74
7.74
7.72
7.72
7.55
7.55
7.53
7.53
7.51
7.51
7.29
7.29
7.27
7.26
7.23
7.21
7.19
7.10
7.08
5.23
2.33
2.31
1.90
1.00
0.98
2.02
0.92
0.98
1.88
2.97
Br
Br
CH3
O
N
O
f1 (ppm)

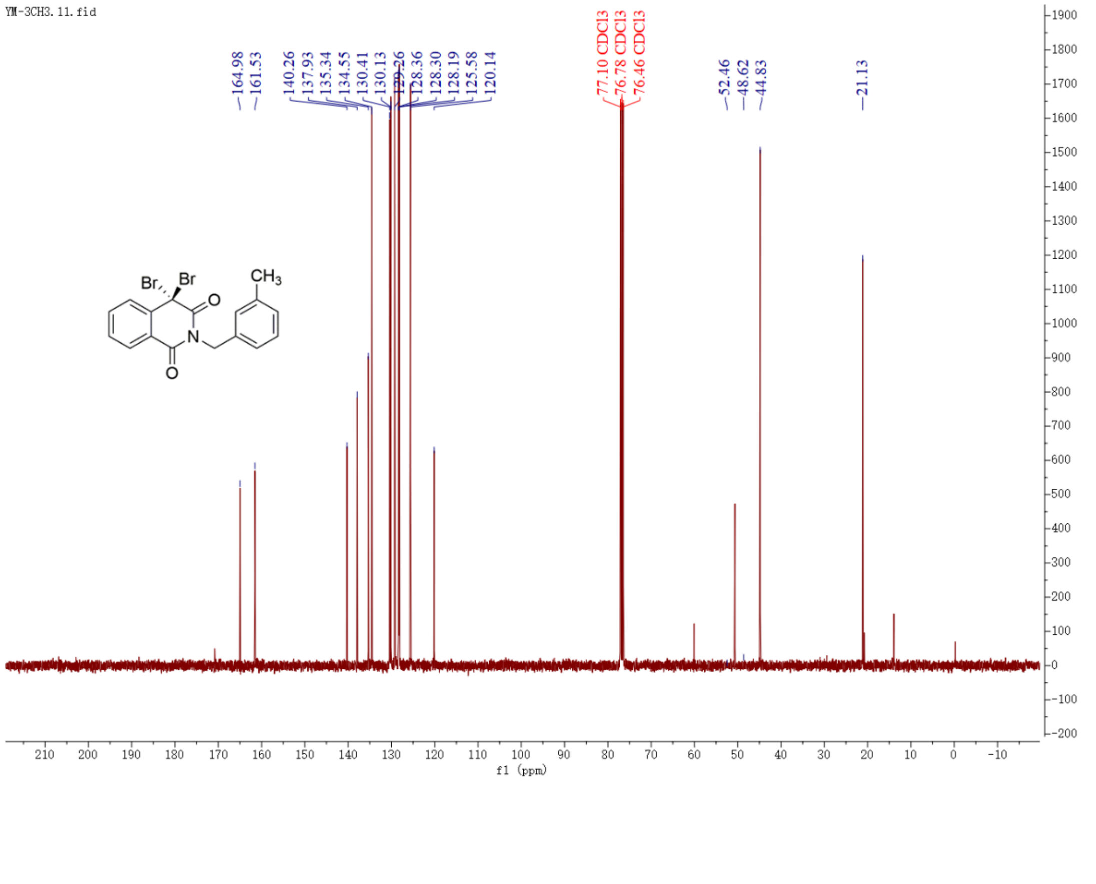
YM-3CH3.11.fid
164.98
161.53
140.26
137.93
135.34
134.55
130.41
130.13
129.26
128.36
128.30
128.19
125.58
120.14
77.10 CDCl3
76.78 CDCl3
76.46 CDCl3
52.46
48.62
44.83
21.13
Br
Br
CH3
O
N
O
f1 (ppm)

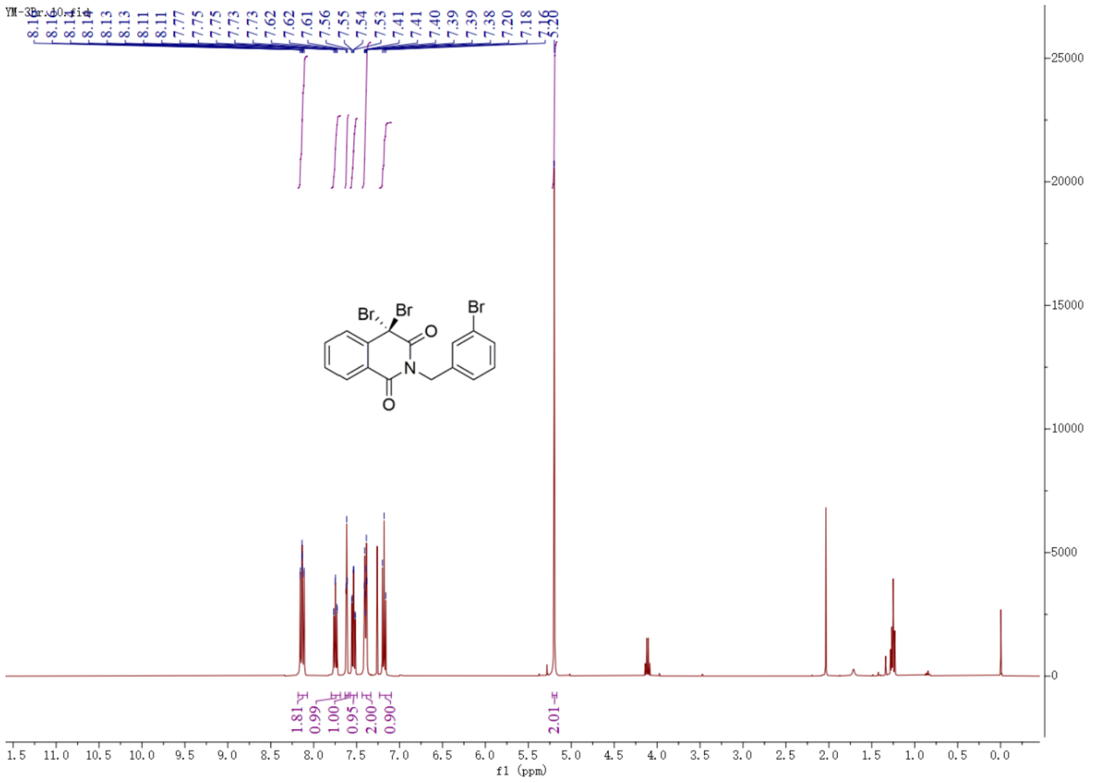
YM-3Br.10.fid
8.14 8.13 8.13 8.11 8.11 7.77 7.75 7.75 7.73 7.73 7.62 7.62 7.61 7.56 7.55 7.54 7.53 7.41 7.41 7.40 7.39 7.39 7.38 7.20 7.18 7.16 5.20
1.81 0.99 1.00 0.95 2.00 0.90 2.01
Br
Br
Br
O
N
O
f1 (ppm)

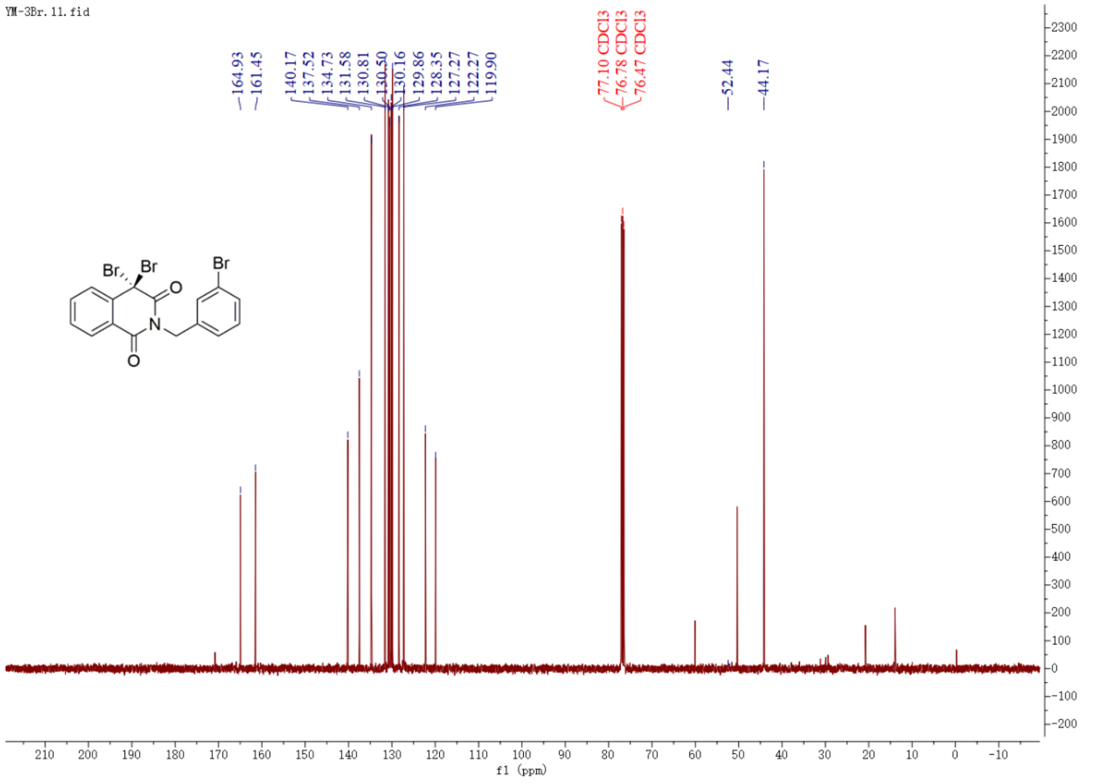
YM-3Br.11.fid
164.93
161.45
140.17 137.52 134.73 131.58 130.81 130.50 130.16 129.86 128.35 127.27 122.27 119.90
77.10 CDCl3
76.78 CDCl3
76.47 CDCl3
52.44
44.17
Br
Br
Br
O
N
O
f1 (ppm)

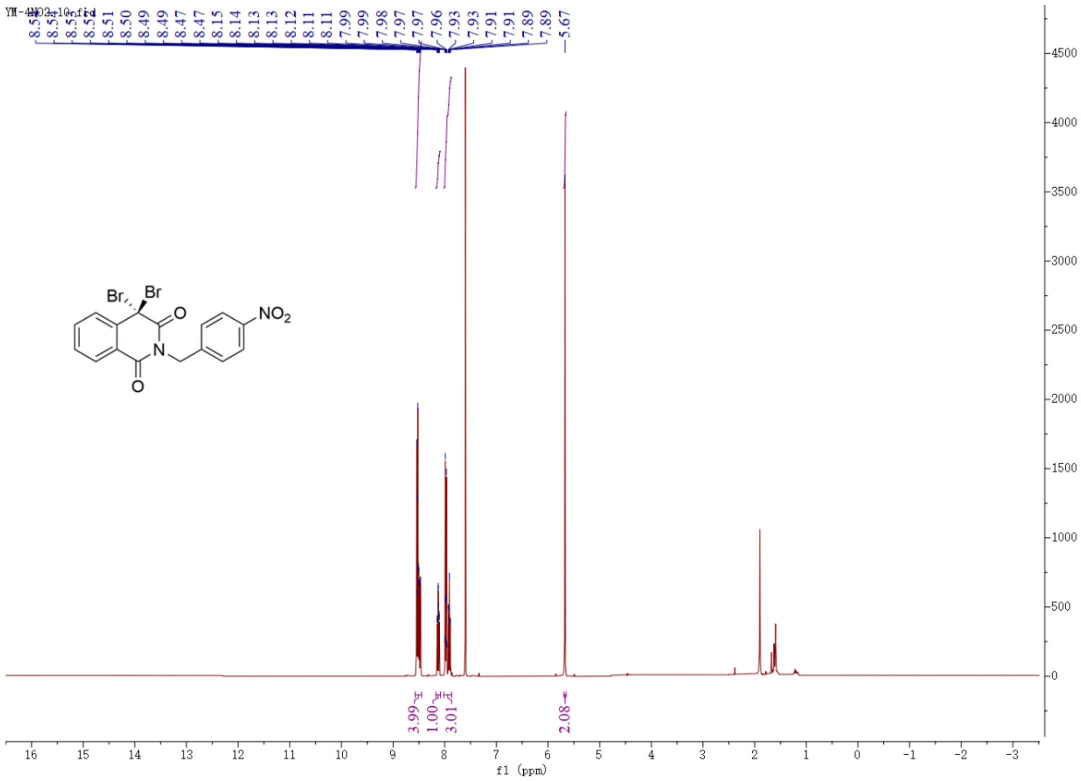
YM-4NO2.10.fid
8.51
8.50
8.49
8.49
8.47
8.47
8.15
8.14
8.13
8.13
8.12
8.11
8.11
7.99
7.99
7.98
7.97
7.97
7.96
7.93
7.93
7.91
7.91
7.89
5.67
Br
Br
O
N
NO2
O
3.99
1.00
3.01
2.08
f1 (ppm)

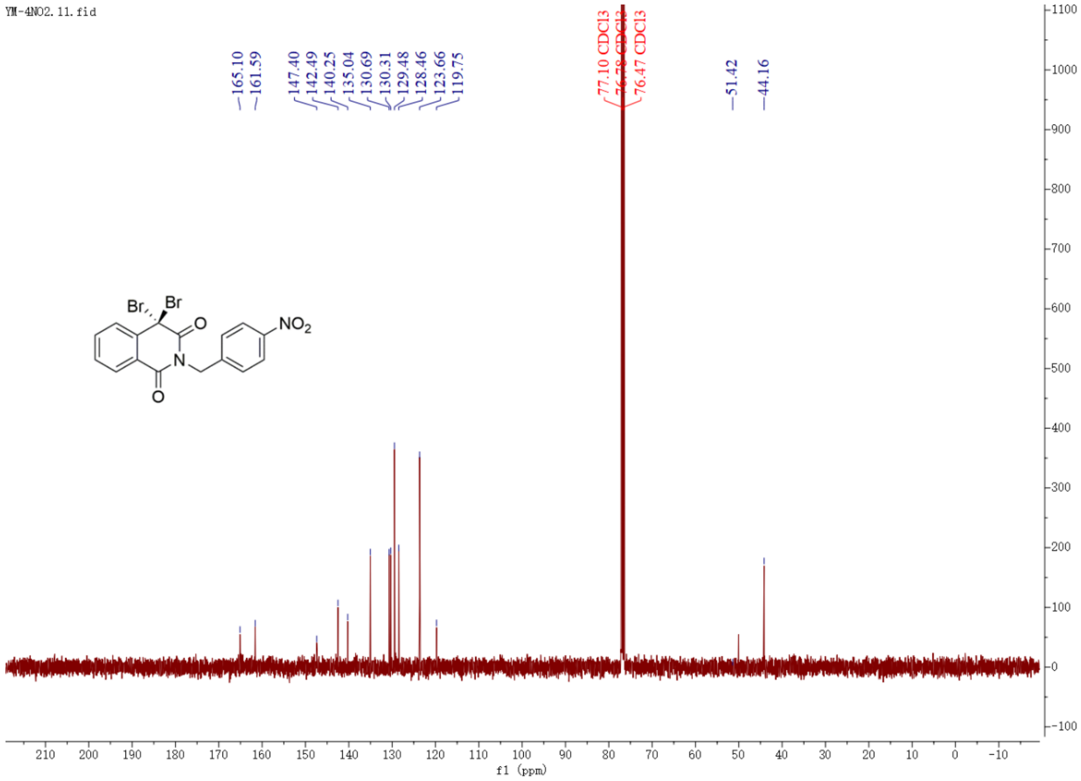
YM-4NO2.11.fid
165.10
161.59
147.40
142.49
140.25
135.04
130.69
130.31
129.48
128.46
123.66
119.75
77.10 CDCl3
76.78 CDCl3
76.47 CDCl3
51.42
44.16
Br
Br
O
N
NO2
O
f1 (ppm)

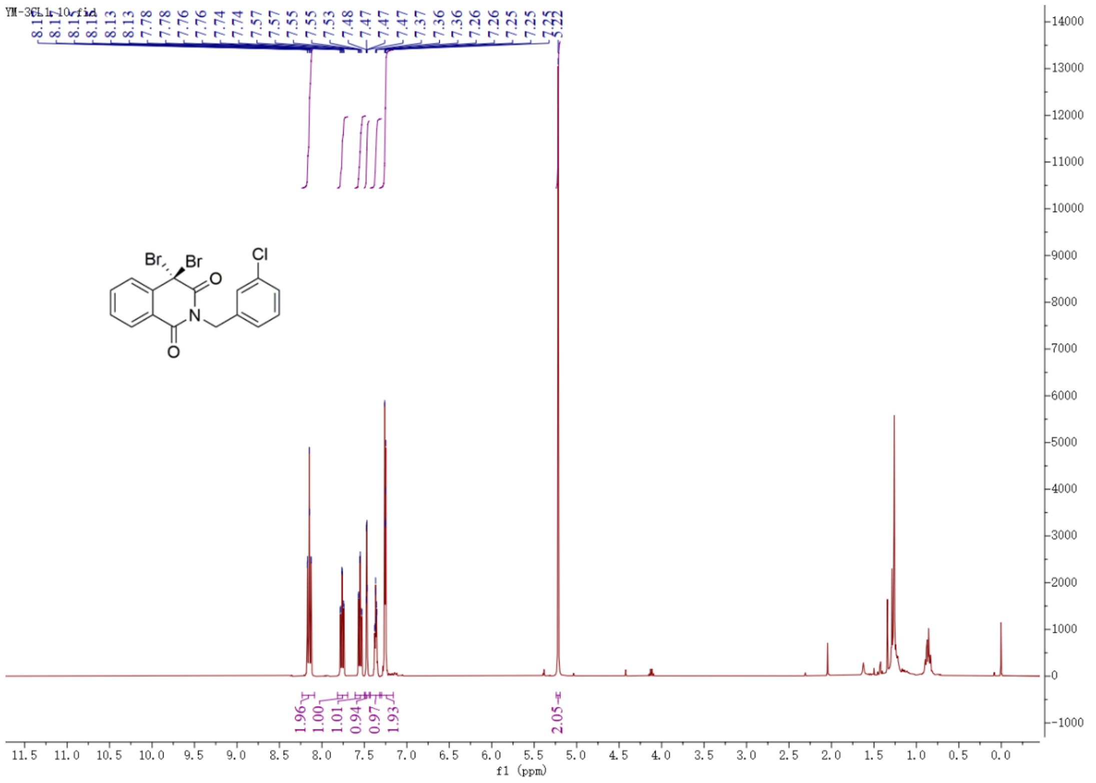
YM-3CL1.10.fid
8.13
8.13
7.78
7.78
7.76
7.76
7.74
7.74
7.57
7.57
7.55
7.55
7.53
7.48
7.47
7.47
7.47
7.37
7.36
7.36
7.26
7.26
7.25
7.25
7.25
5.22
1.96
1.00
1.01
0.94
0.97
1.93
2.05
f1 (ppm)

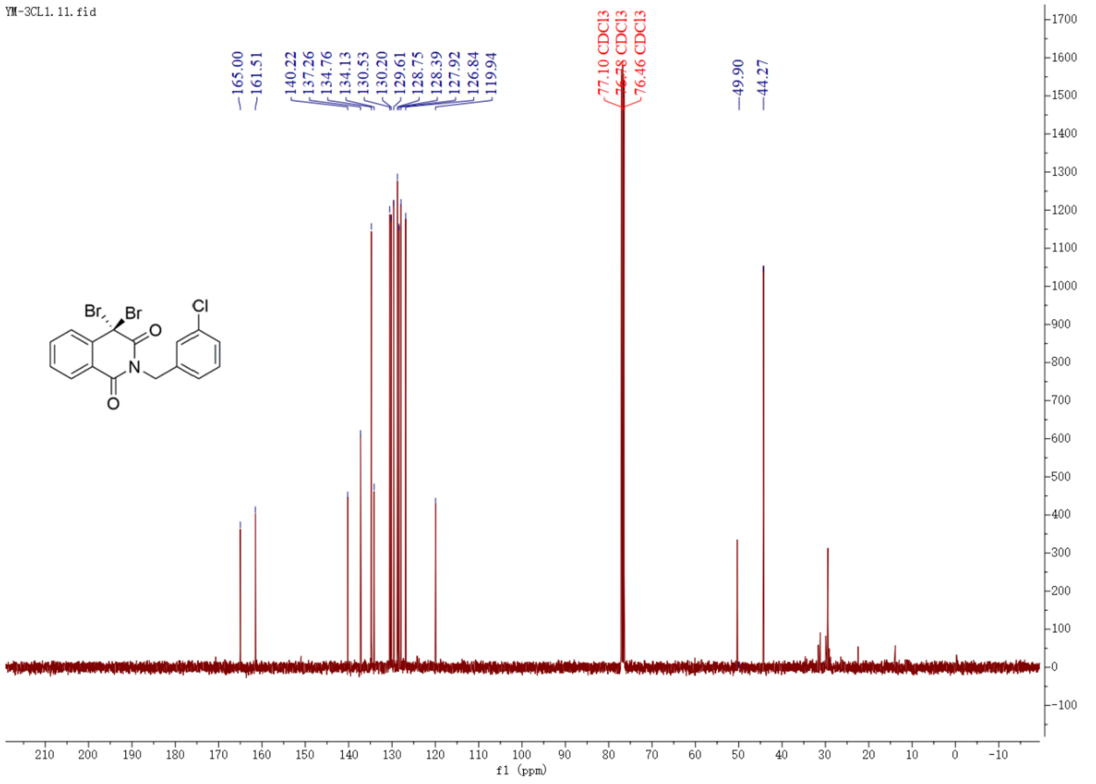
YM-3CL1.11.fid
165.00
161.51
140.22
137.26
134.76
134.13
130.53
130.20
129.61
128.75
128.39
127.92
126.84
119.94
77.10 CDCl3
76.78 CDCl3
76.46 CDCl3
49.90
44.27
f1 (ppm)

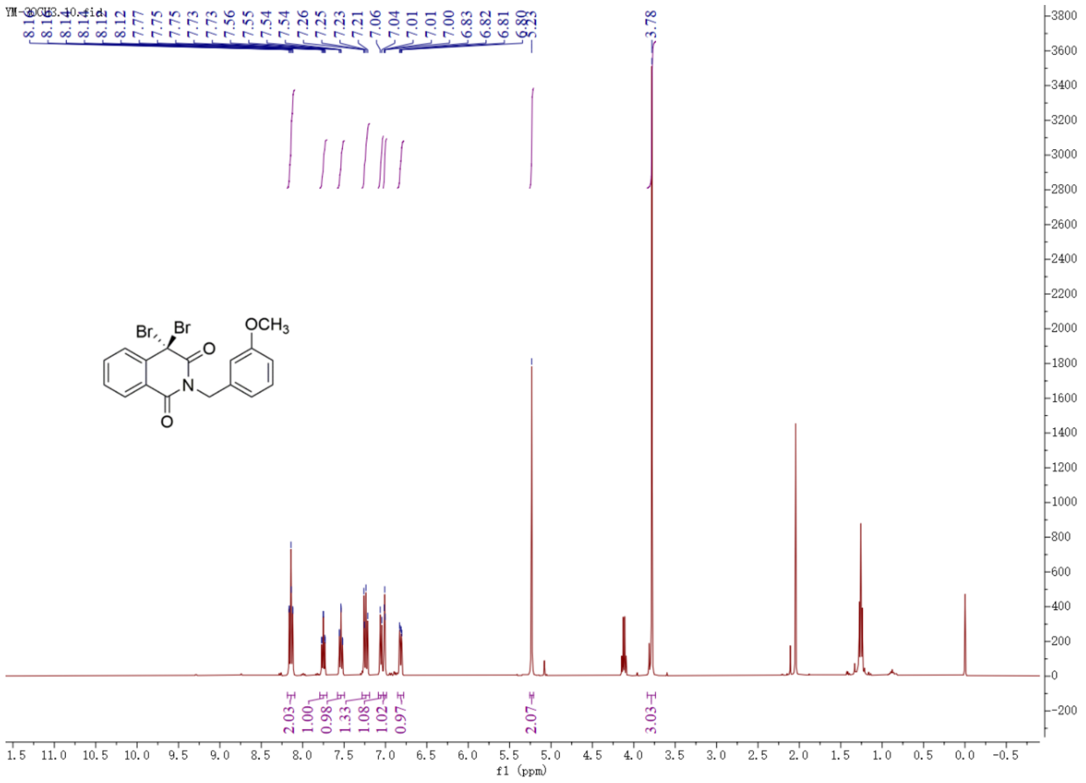

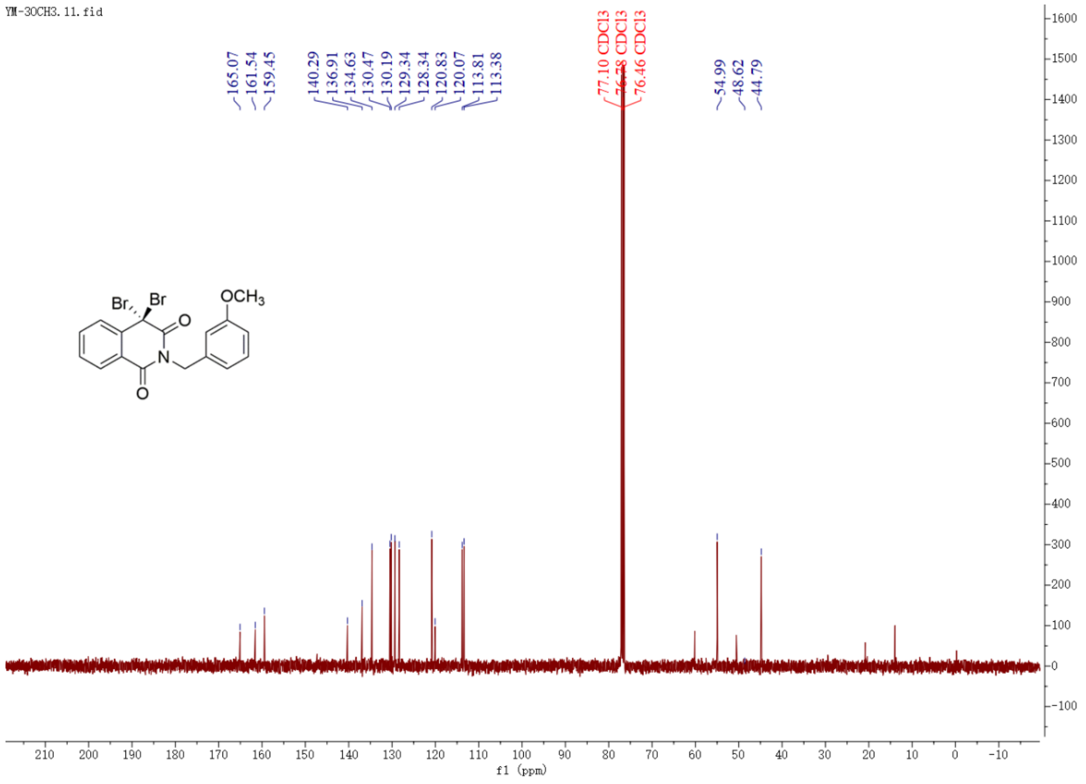

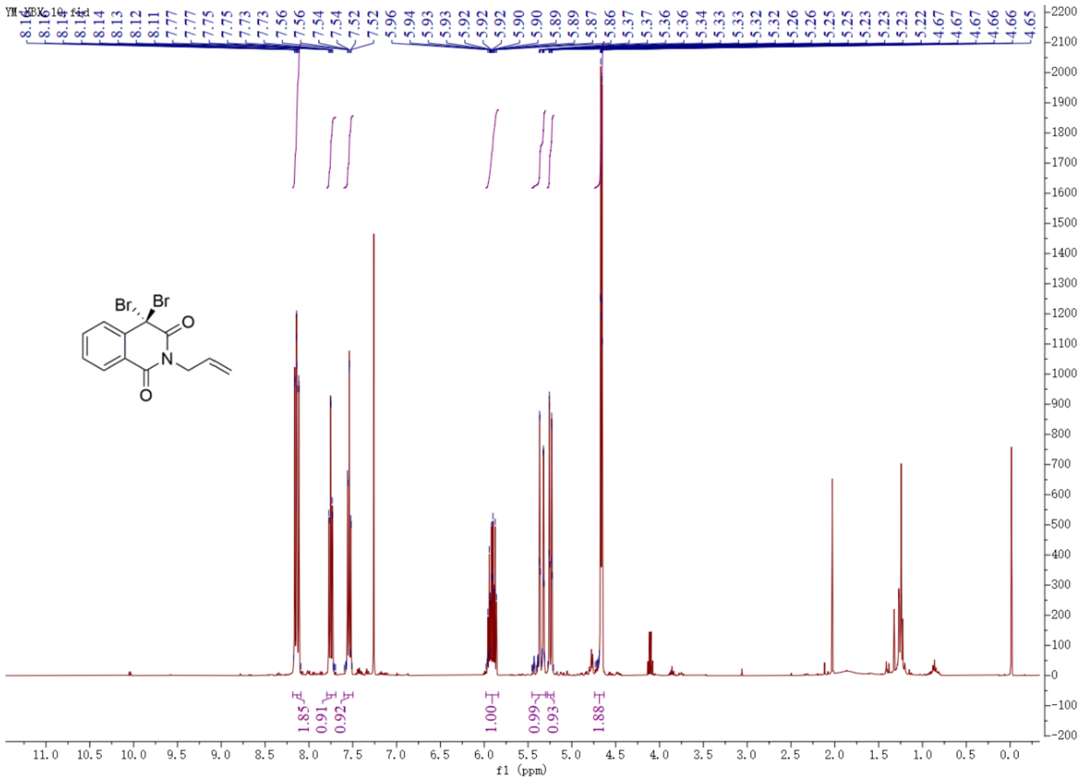
YM-XBX.10.fid
1.85
0.91
0.92
1.00
0.99
0.93
1.88
f1 (ppm)

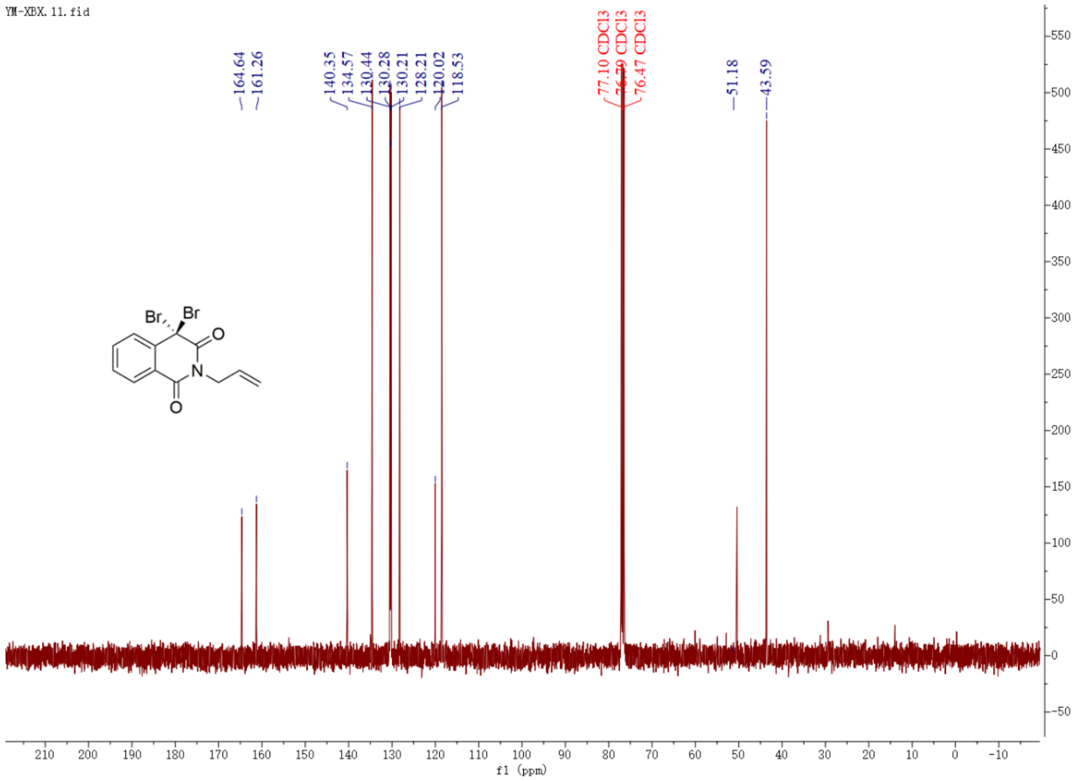
YM-XBX.11.fid
164.64
161.26
140.35
134.57
130.44
130.28
130.21
128.21
120.02
118.53
77.10 CDCl3
76.79 CDCl3
76.47 CDCl3
51.18
43.59
f1 (ppm)

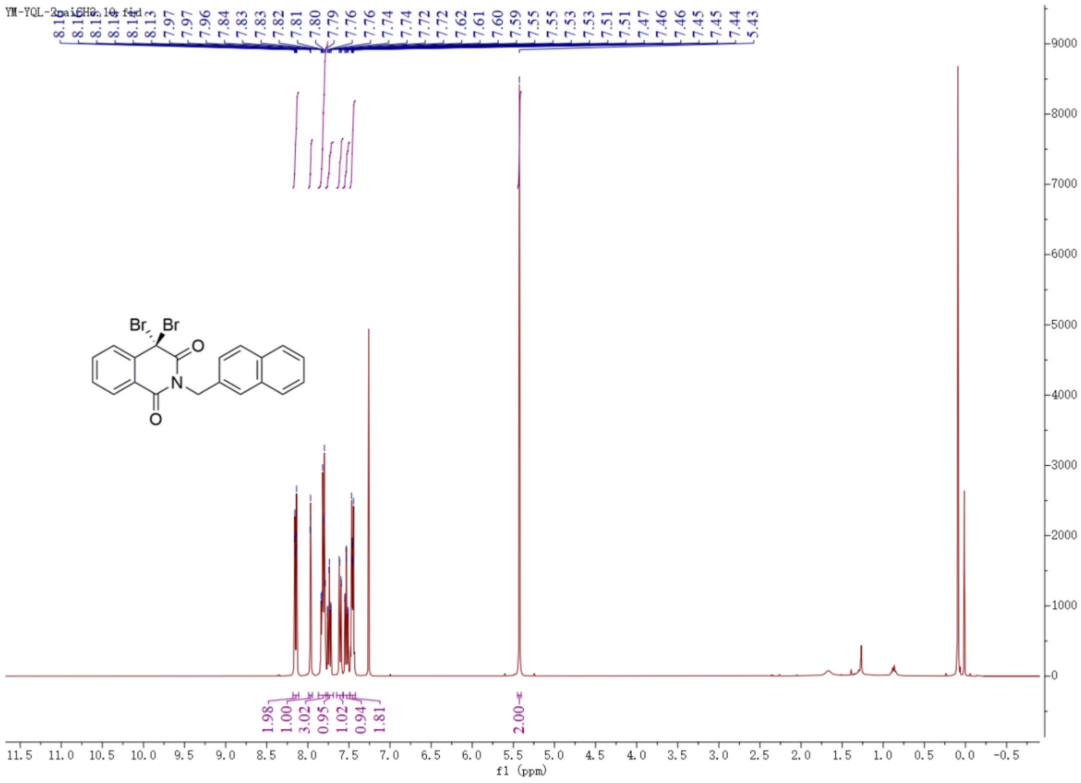

8.13
7.97
7.97
7.96
7.84
7.83
7.83
7.82
7.81
7.80
7.79
7.76
7.76
7.74
7.74
7.72
7.72
7.62
7.61
7.60
7.59
7.55
7.55
7.53
7.53
7.51
7.51
7.47
7.46
7.46
7.45
7.45
7.44
5.43
Br
Br
O
N
O
1.98
1.00
3.02
0.95
1.02
0.94
1.81
2.00
f1 (ppm)

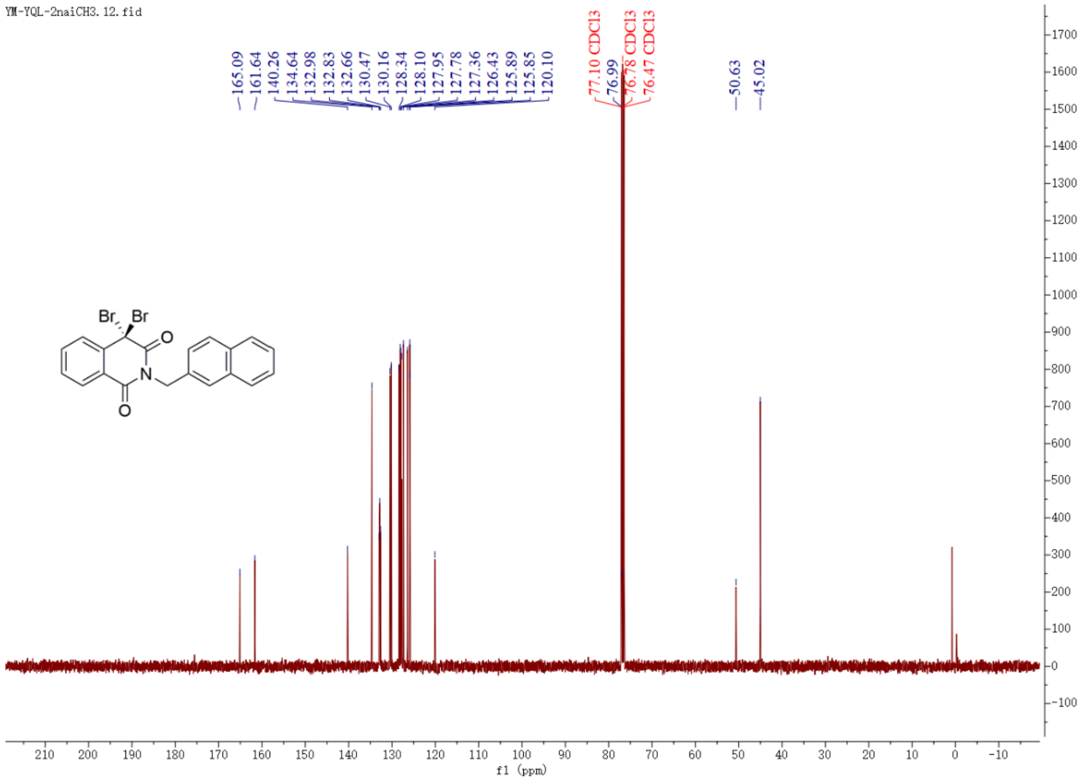

YM-YQL-2naiCH3.12.fid
165.09
161.64
140.26
134.64
132.98
132.83
132.66
130.47
130.16
128.34
128.10
127.95
127.78
127.36
126.43
125.89
125.85
120.10
77.10 CDCl3
76.99
76.78 CDCl3
76.47 CDCl3
50.63
45.02
Br
Br
O
N
O
f1 (ppm)

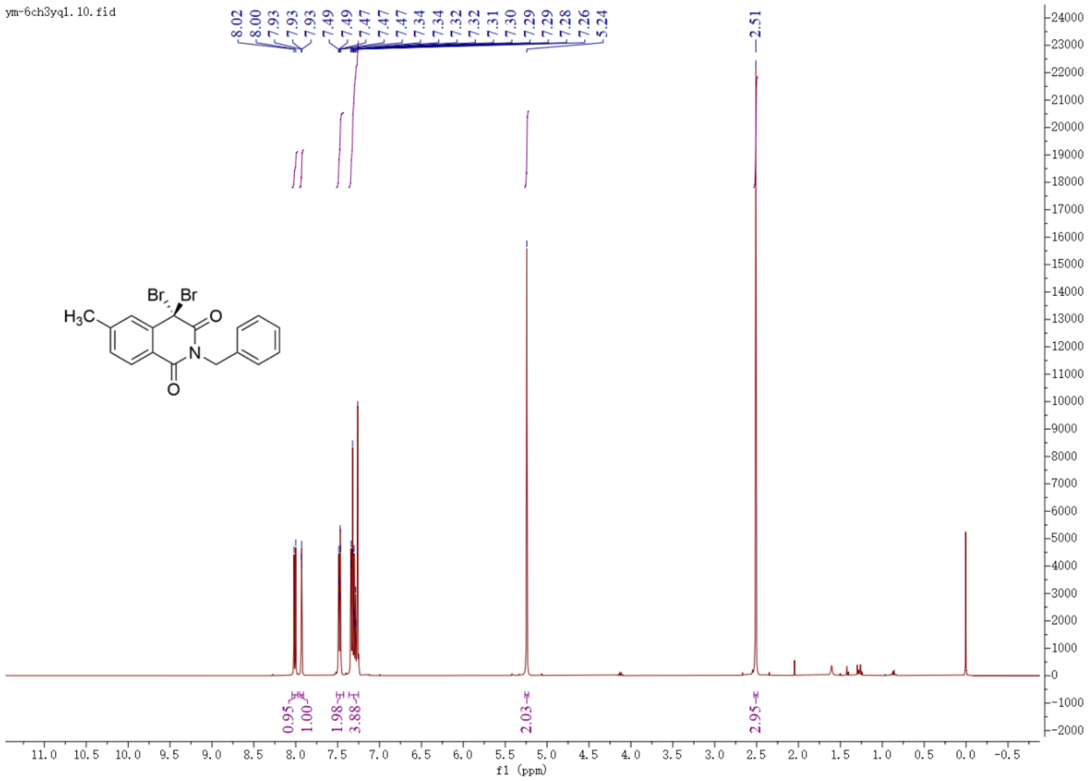
ym-6ch3yql.10.fid
8.02 8.00 7.93 7.93 7.93 7.49 7.49 7.47 7.47 7.47 7.34 7.32 7.32 7.31 7.30 7.29 7.29 7.28 7.26 5.24
2.51
0.95 1.00 1.98 3.88 2.03 2.95
f1 (ppm)
H3C
Br
Br
O
N
O

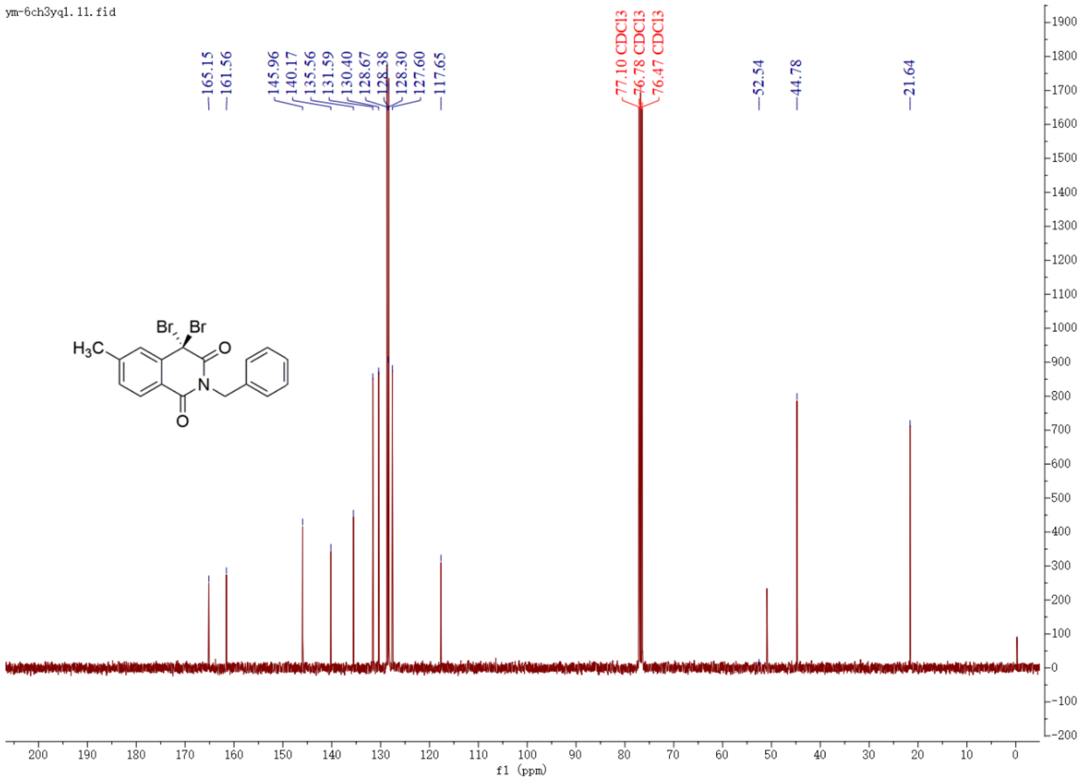
ym-6ch3yql.11.fid
165.15 161.56
145.96 140.17 135.56 131.59 130.40 128.67 128.38 128.30 127.60 117.65
77.10 CDCl3 76.78 CDCl3 76.47 CDCl3
52.54 44.78 21.64
f1 (ppm)
H3C
Br
Br
O
N
O

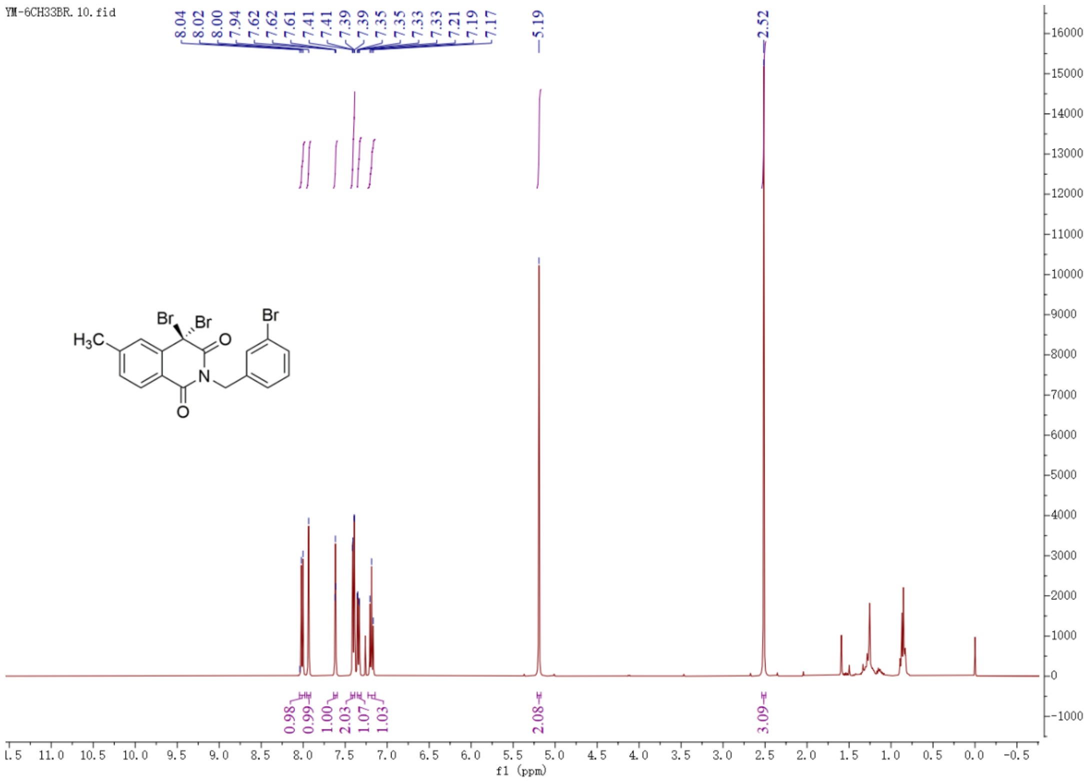
YM-6CH33BR.10.fid
8.04
8.02
8.00
7.94
7.62
7.62
7.61
7.41
7.41
7.39
7.39
7.35
7.35
7.33
7.33
7.21
7.19
7.17
5.19
2.52
0.98
0.99
1.00
2.03
1.07
1.03
2.08
3.09
Br
Br
Br
H3C
O
N
O
f1 (ppm)

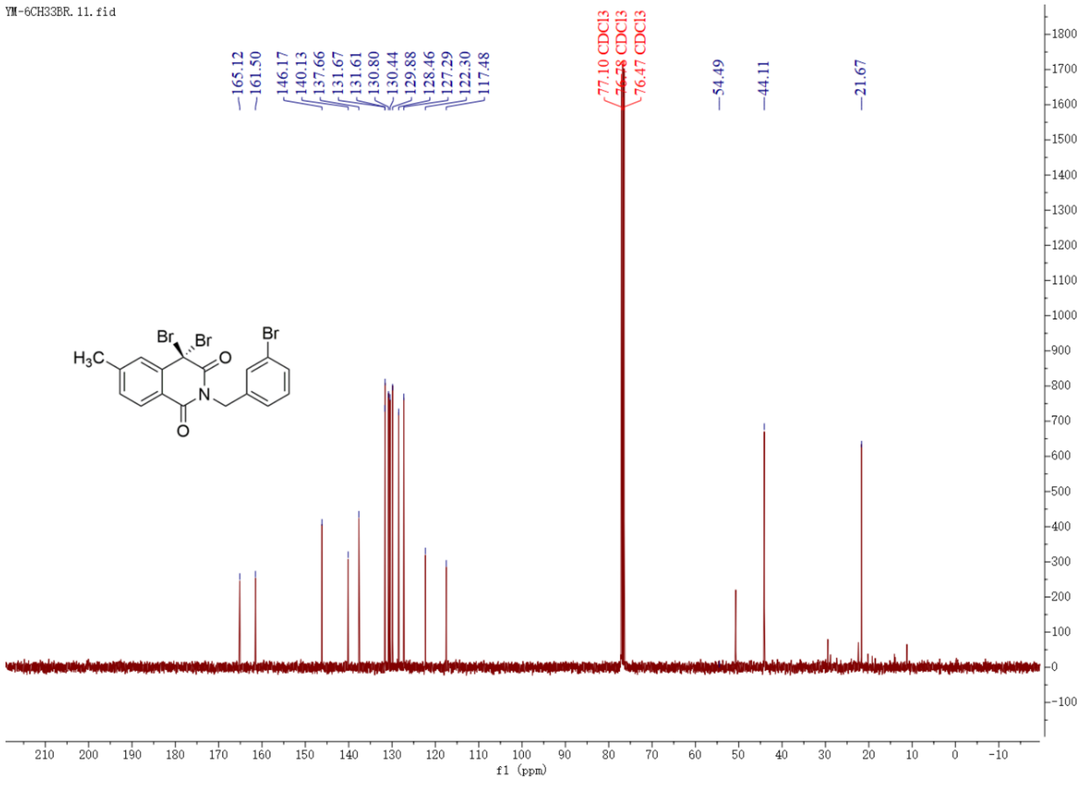
YM-6CH33BR.11.fid
165.12
161.50
146.17
140.13
137.66
131.67
131.61
130.80
130.44
129.88
128.46
127.29
122.30
117.48
77.10 CDCl3
76.78 CDCl3
76.47 CDCl3
54.49
44.11
21.67
Br
Br
Br
H3C
O
N
O
f1 (ppm)

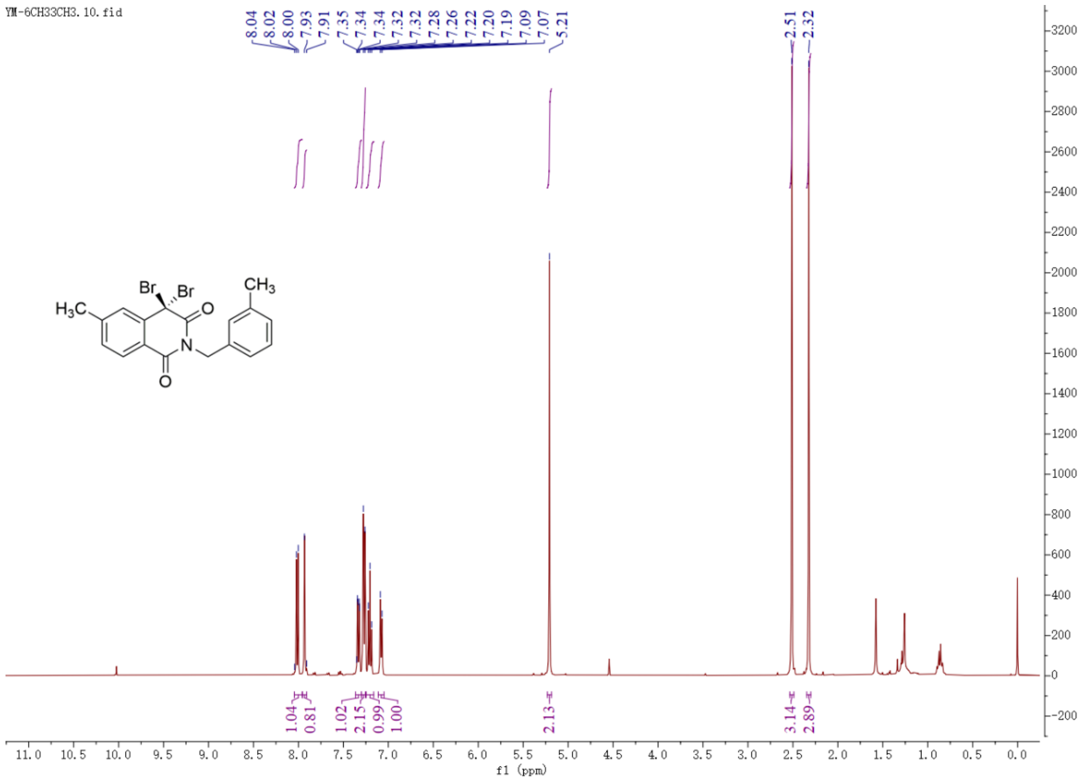
YM-6CH33CH3.10.fid
8.04
8.02
8.00
7.93
7.91
7.35
7.34
7.34
7.32
7.32
7.28
7.26
7.22
7.20
7.19
7.09
7.07
5.21
2.51
2.32
1.04
0.81
1.02
2.15
0.99
1.00
2.13
3.14
2.89
f1 (ppm)

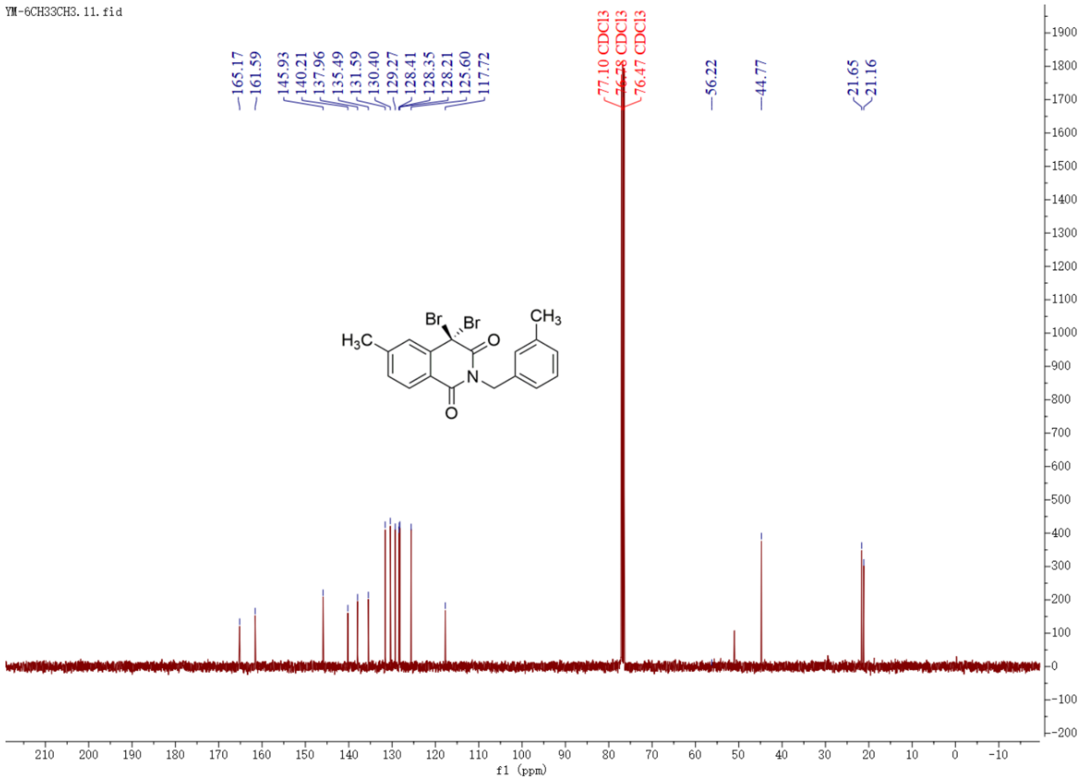
YM-6CH33CH3.11.fid
165.17
161.59
145.93
140.21
137.96
135.49
131.59
130.40
129.27
128.41
128.35
128.21
125.60
117.72
77.10 CDCl3
76.78 CDCl3
76.47 CDCl3
56.22
44.77
21.65
21.16
f1 (ppm)

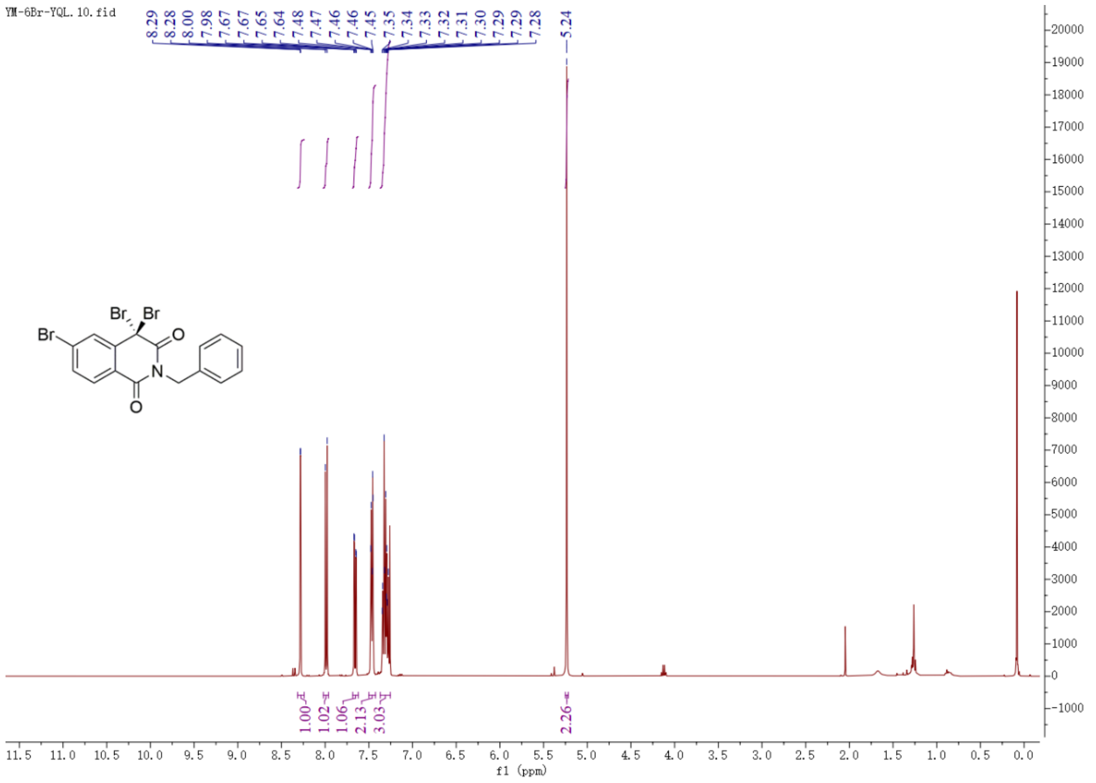
YM-6Br-YQL.10.fid
8.29
8.28
8.00
7.98
7.67
7.67
7.65
7.64
7.48
7.47
7.46
7.46
7.45
7.35
7.34
7.33
7.32
7.31
7.30
7.29
7.29
7.28
5.24
Br
Br
Br
O
N
O
1.00
1.02
1.06
2.13
3.03
2.26
f1 (ppm)

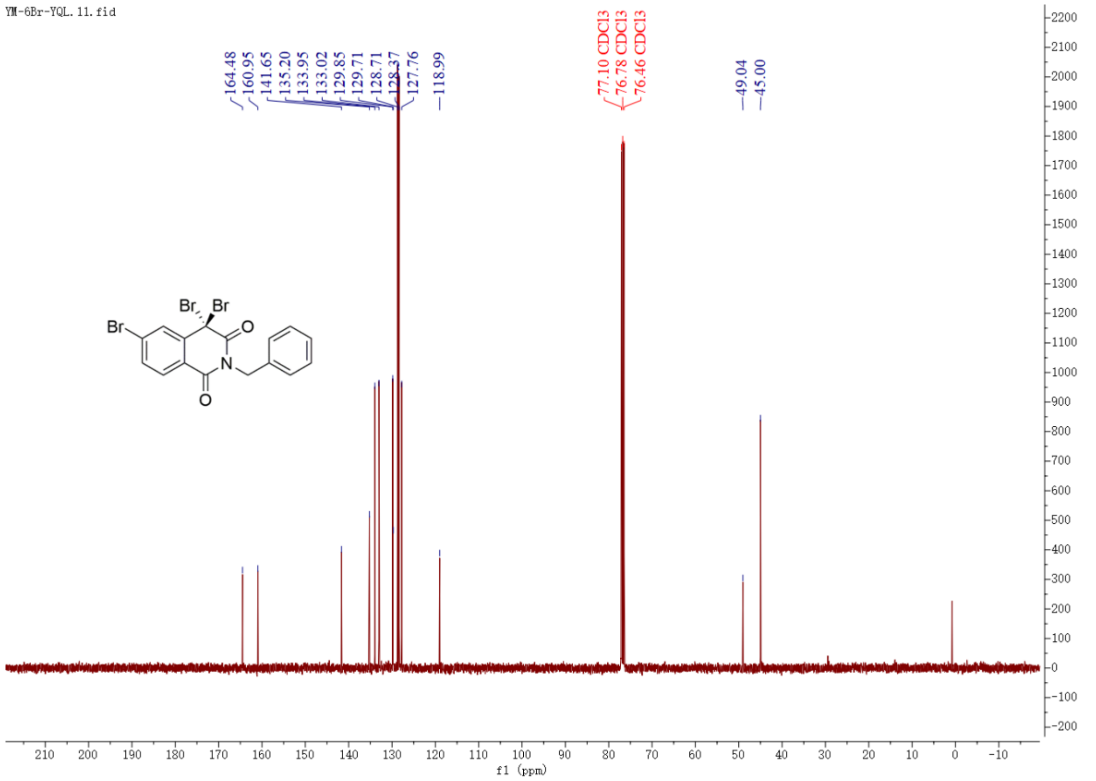
YM-6Br-YQL.11.fid
164.48
160.95
141.65
135.20
133.95
133.02
129.85
129.71
128.71
128.37
127.76
118.99
77.10 CDCl3
76.78 CDCl3
76.46 CDCl3
49.04
45.00
Br
Br
Br
O
N
O
f1 (ppm)

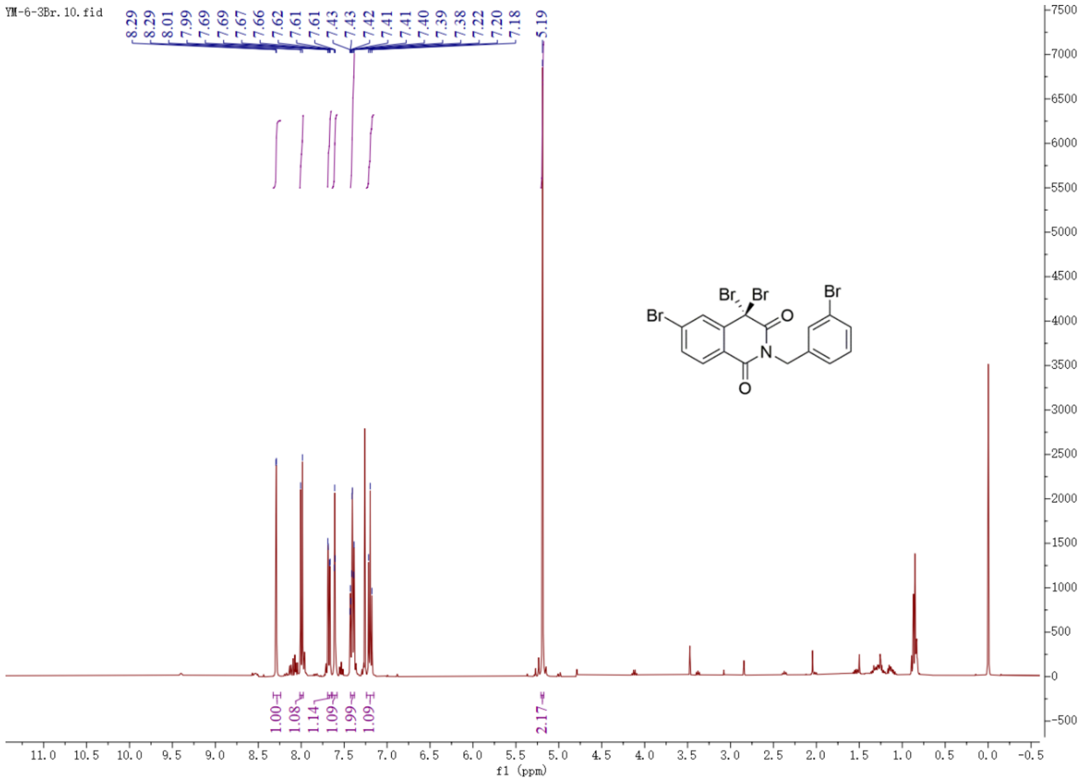
YM-6-3Br.10.fid
8.29
8.29
8.01
7.99
7.69
7.69
7.67
7.66
7.62
7.61
7.61
7.43
7.43
7.42
7.41
7.40
7.39
7.38
7.22
7.20
7.18
5.19
Br
Br
Br
Br
O
N
O
1.00
1.08
1.14
1.09
1.99
1.09
2.17
f1 (ppm)

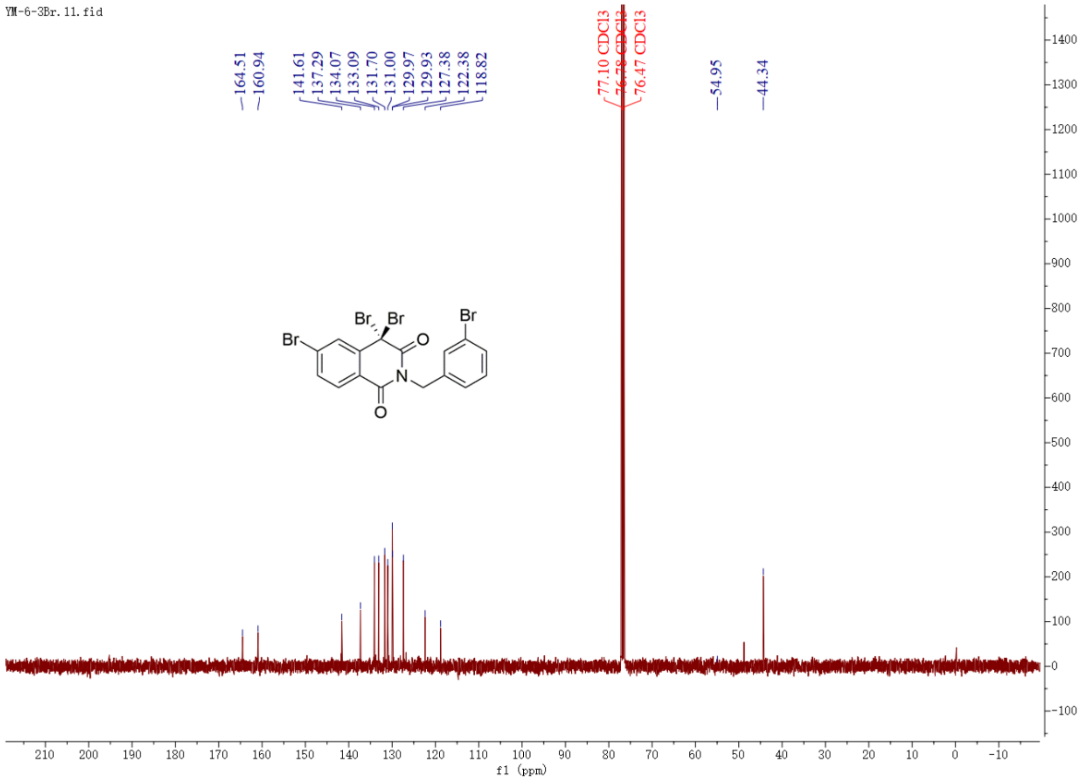
YM-6-3Br.11.fid
164.51
160.94
141.61
137.29
134.07
133.09
131.70
131.00
129.97
129.93
127.38
122.38
118.82
77.10 CDCl3
76.78 CDCl3
76.47 CDCl3
54.95
44.34
Br
Br
Br
Br
O
N
O
f1 (ppm)

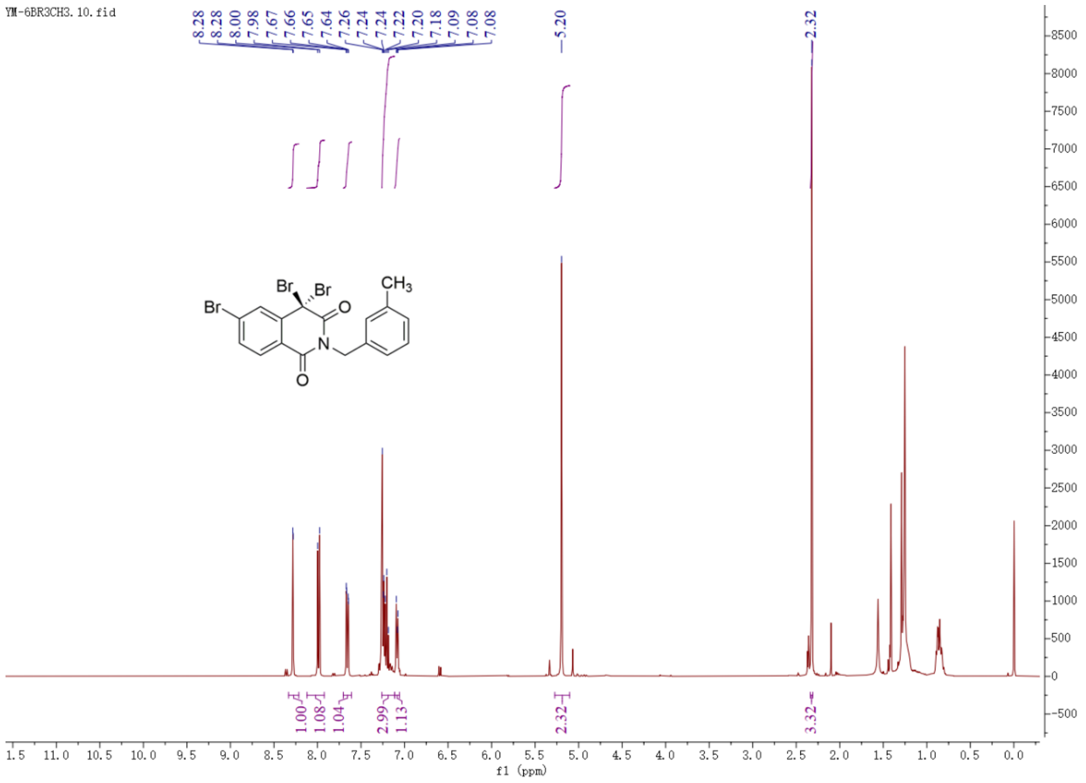
YM-6BR3CH3.10.fid
8.28
8.28
8.00
7.98
7.67
7.66
7.65
7.64
7.26
7.24
7.22
7.20
7.18
7.09
7.08
7.08
5.20
2.32
1.00
1.08
1.04
2.99
1.13
2.32
3.32
Br
Br
Br
O
N
O
CH3
f1 (ppm)

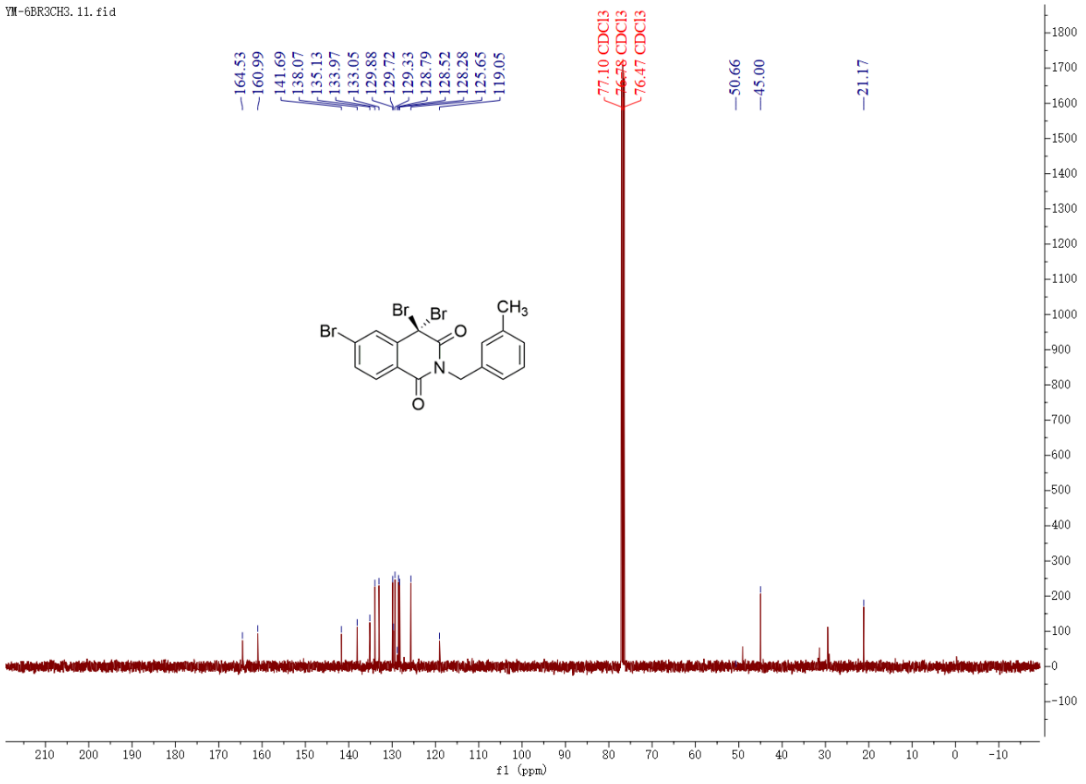
YM-6BR3CH3.11.fid
164.53
160.99
141.69
138.07
135.13
133.97
133.05
129.88
129.72
129.33
128.79
128.52
128.28
125.65
119.05
77.10 CDCl3
76.78 CDCl3
76.47 CDCl3
50.66
45.00
21.17
Br
Br
Br
O
N
O
CH3
f1 (ppm)

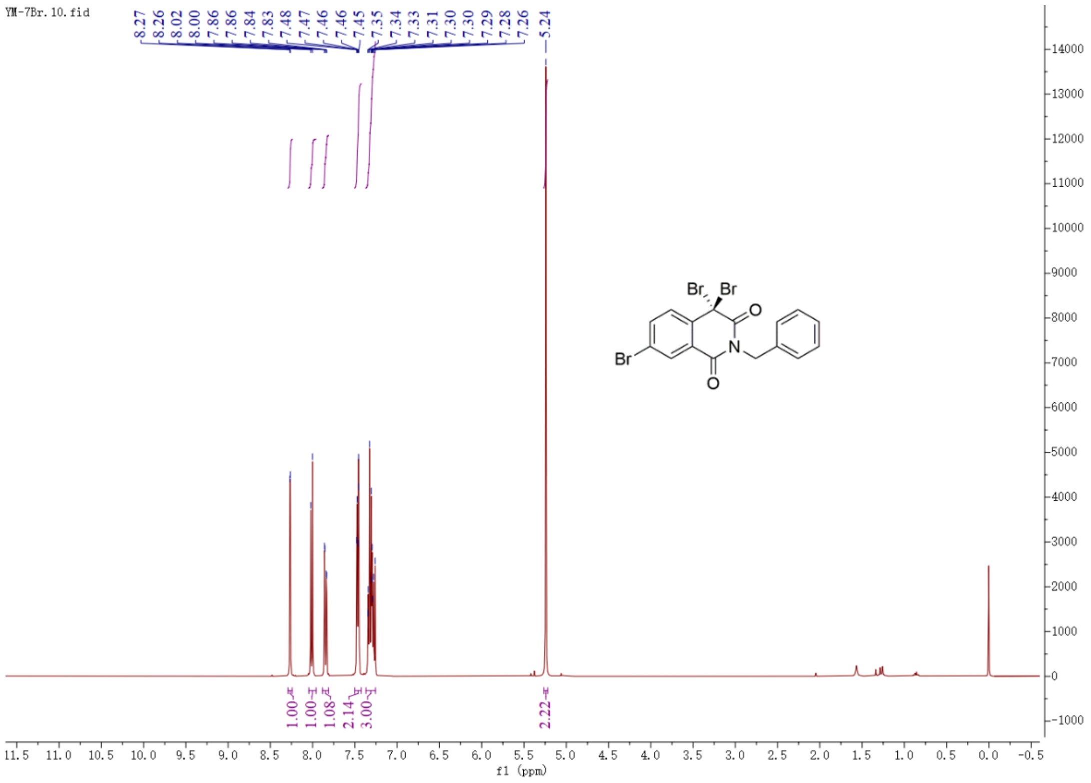
YM-7Br.10.fid
8.27
8.26
8.02
8.00
7.86
7.86
7.84
7.83
7.48
7.47
7.46
7.46
7.45
7.35
7.34
7.33
7.31
7.30
7.30
7.29
7.28
7.26
5.24
1.00
1.00
1.08
2.14
3.00
2.22
Br
Br
Br
O
N
O
f1 (ppm)

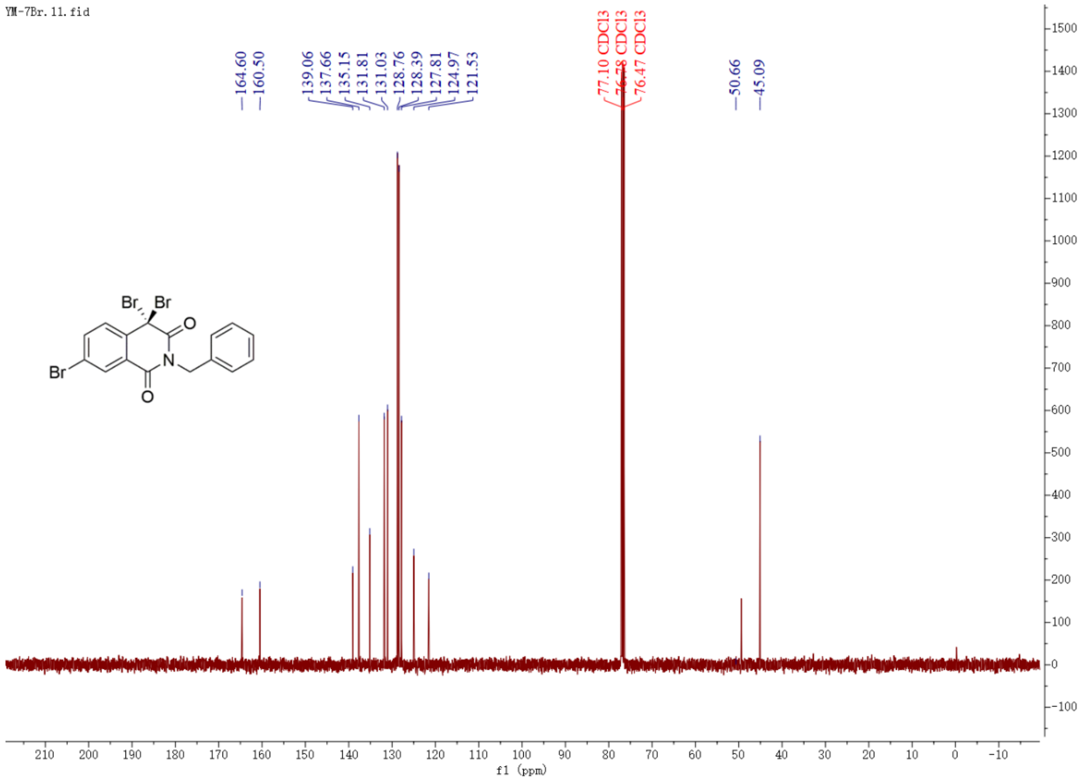
YM-7Br.11.fid
164.60
160.50
139.06
137.66
135.15
131.81
131.03
128.76
128.39
127.81
124.97
121.53
77.10 CDCl3
76.78 CDCl3
76.47 CDCl3
50.66
45.09
Br
Br
Br
O
N
O
f1 (ppm)

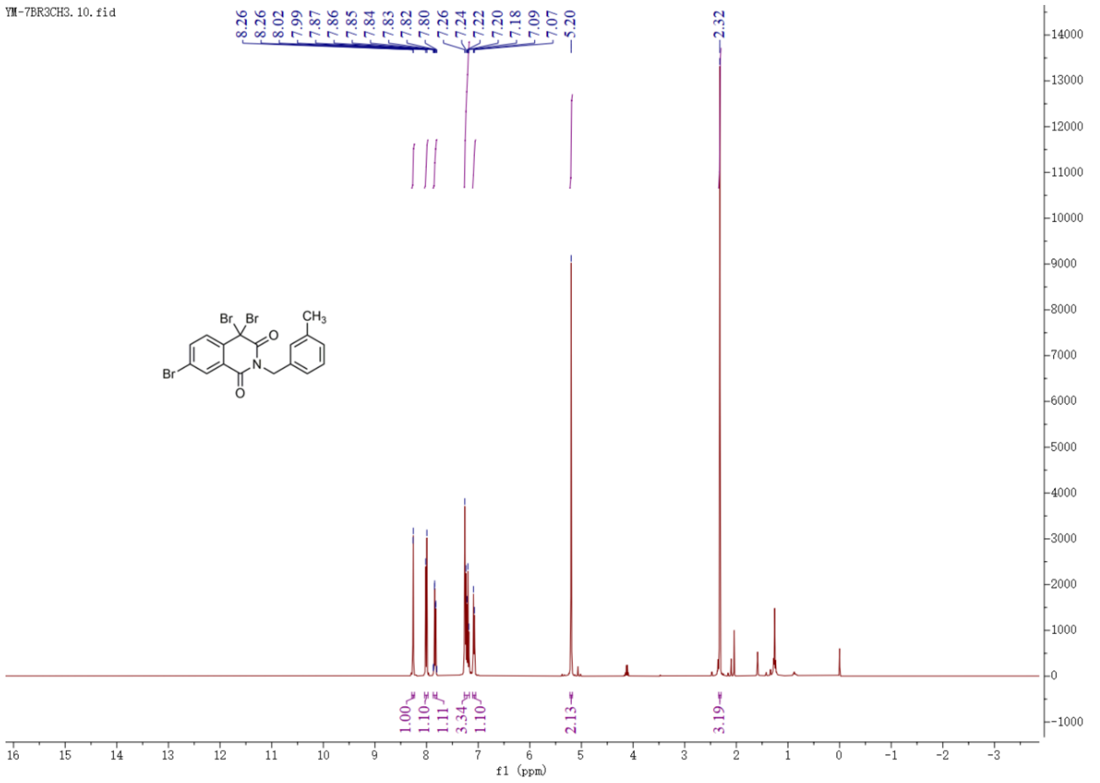
YM-7BR3CH3.10.fid
8.26 8.26 8.02 7.99 7.87 7.86 7.85 7.84 7.83 7.82 7.80 7.26 7.24 7.22 7.20 7.18 7.09 7.07
5.20
2.32
1.00 1.10 1.11 3.34 1.10
2.13
3.19
Br Br CH3 O N Br O
f1 (ppm)

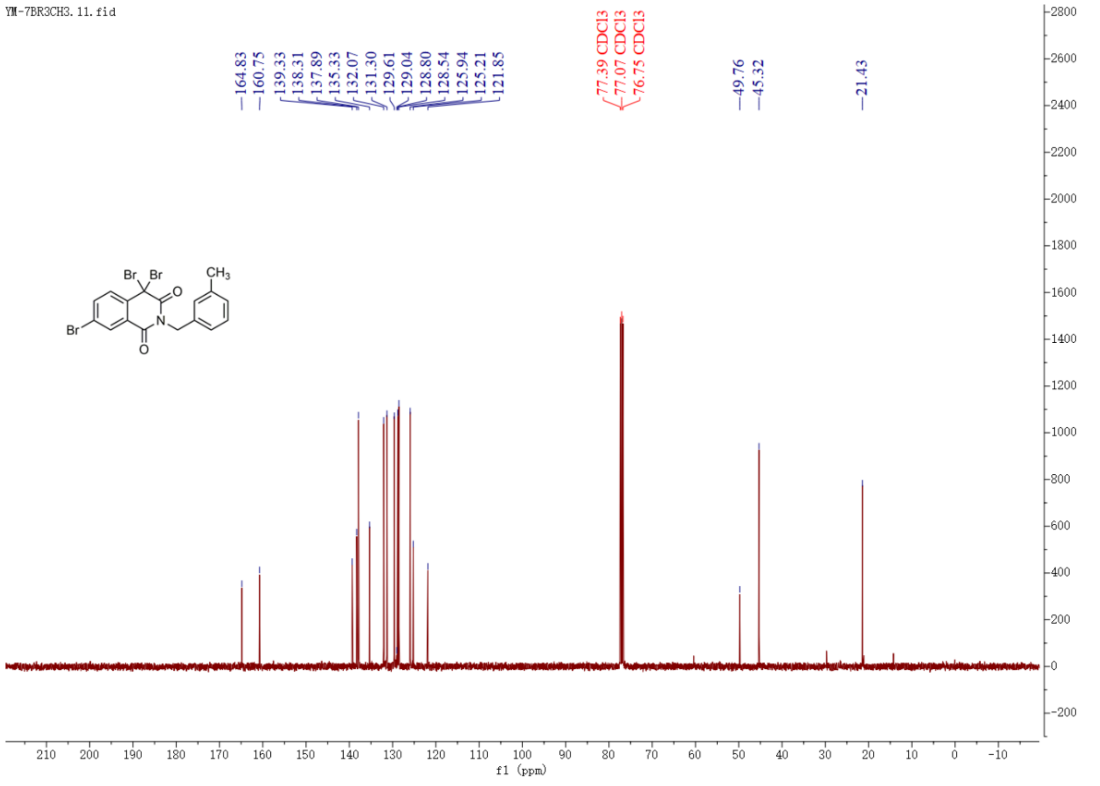
YM-7BR3CH3.11.fid
164.83 160.75 139.33 138.31 137.89 135.33 132.07 131.30 129.61 129.04 128.80 128.54 125.94 125.21 121.85
77.39 CDCl3 77.07 CDCl3 76.75 CDCl3
49.76 45.32
21.43
Br Br CH3 O N Br O
f1 (ppm)

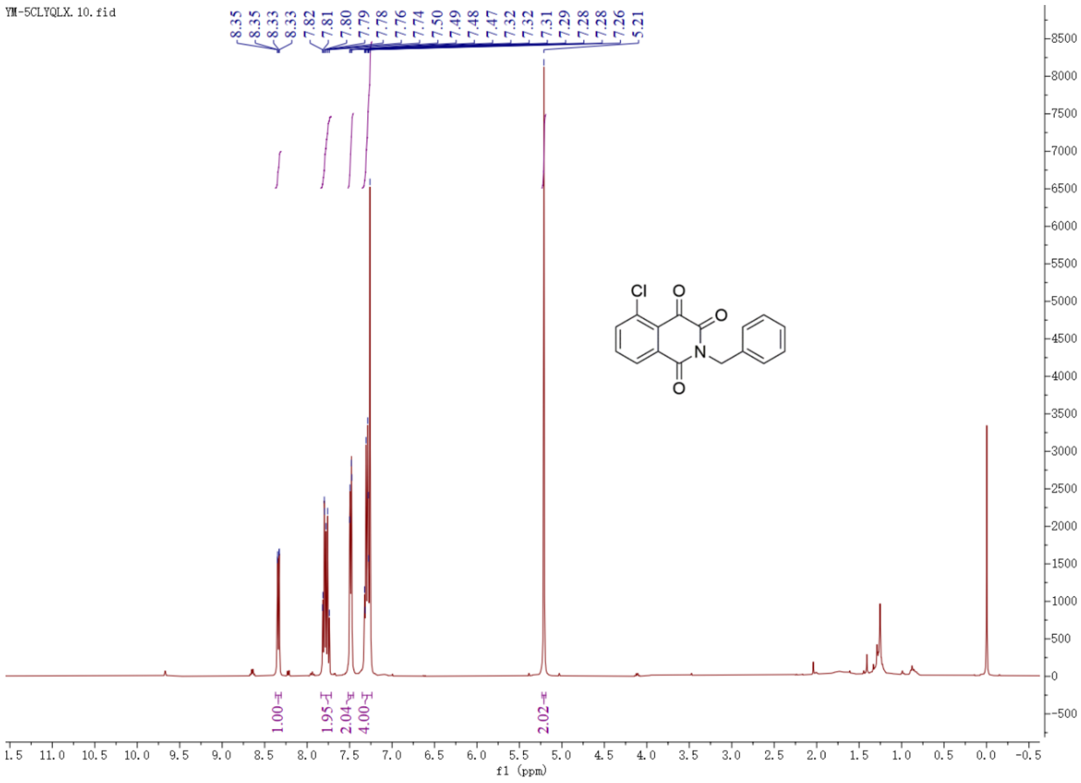
YM-5CLYQLX.10.fid
8.35 8.35 8.33 8.33 7.82 7.81 7.80 7.79 7.78 7.76 7.74 7.50 7.49 7.48 7.47 7.32 7.32 7.31 7.29 7.28 7.28 7.26 5.21
1.00 1.95 2.04 4.00 2.02
Cl
O
N
f1 (ppm)

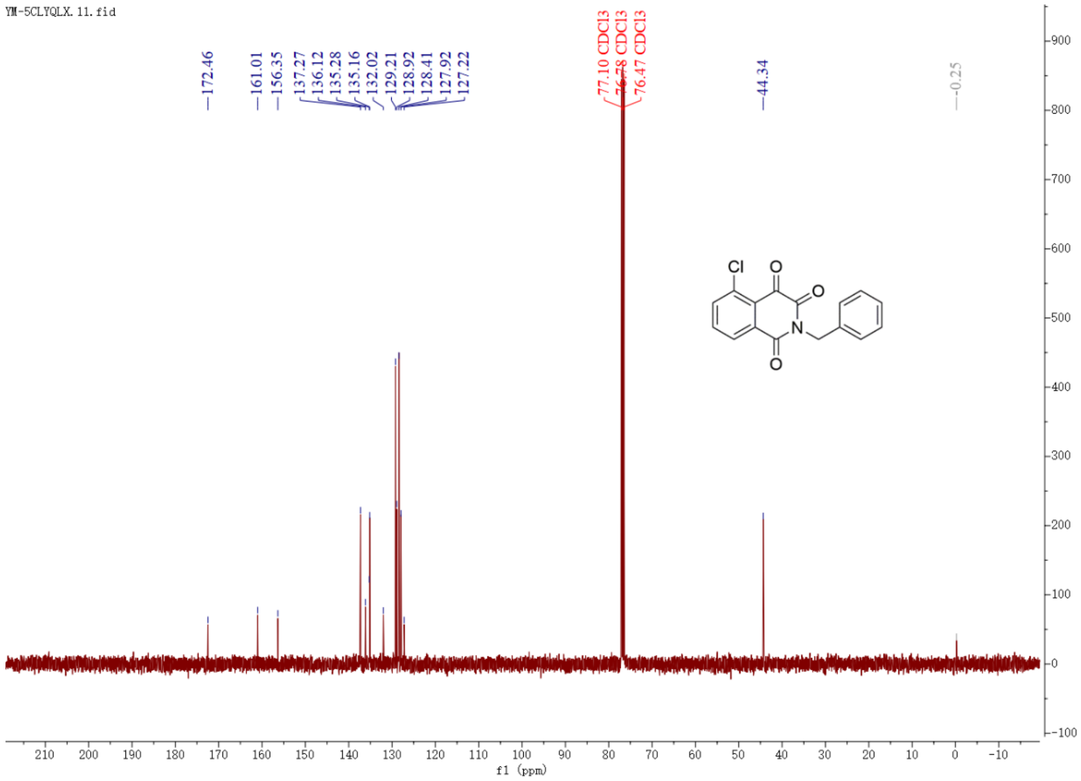
YM-5CLYQLX.11.fid
172.46
161.01
156.35
137.27 136.12 135.28 135.16 132.02 129.21 128.92 128.41 127.92 127.22
77.10 CDCl3
76.78 CDCl3
76.47 CDCl3
44.34
-0.25
Cl
O
N
f1 (ppm)

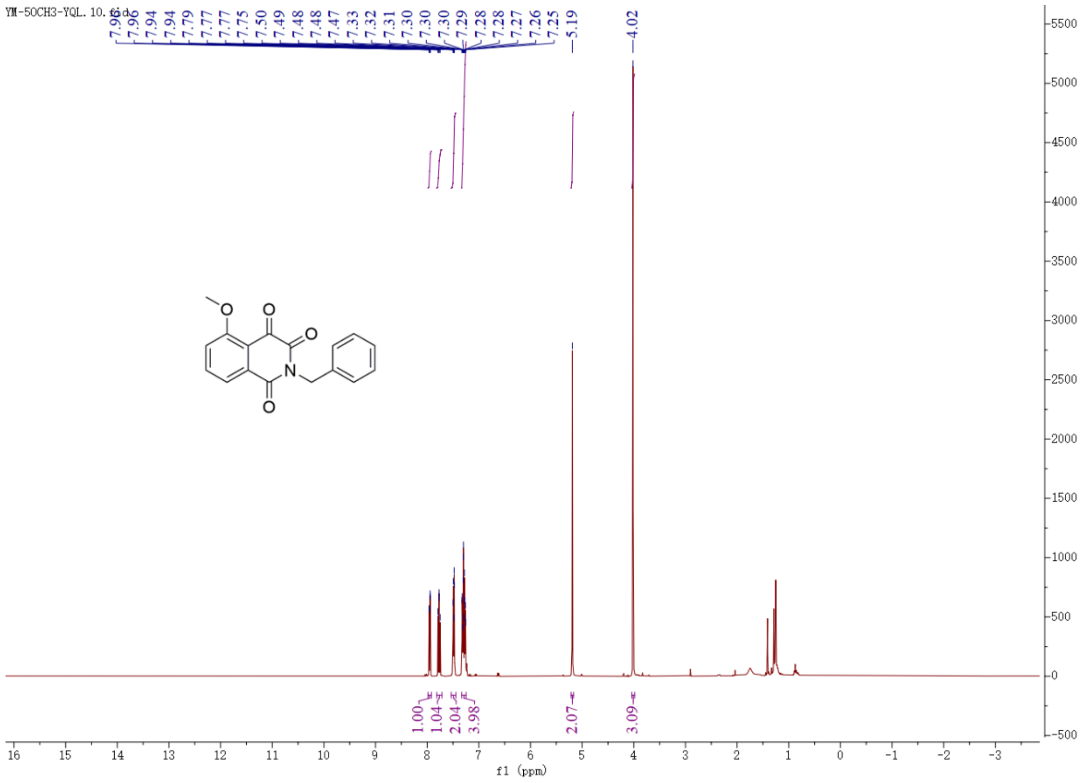
YM-5OCH3-YQL.10.fid
7.96
7.96
7.94
7.94
7.79
7.77
7.77
7.75
7.50
7.49
7.48
7.48
7.47
7.33
7.32
7.31
7.30
7.30
7.30
7.29
7.28
7.28
7.27
7.26
7.25
5.19
4.02
1.00
1.04
2.04
3.98
2.07
3.09
f1 (ppm)

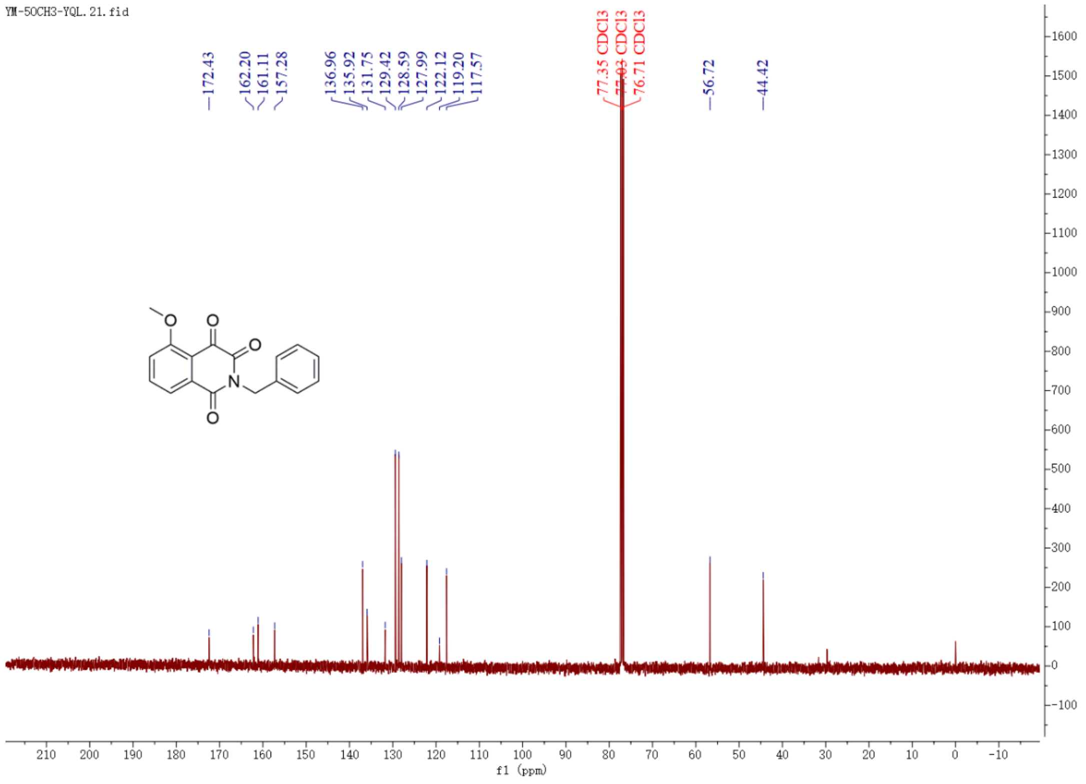
YM-5OCH3-YQL.21.fid
172.43
162.20
161.11
157.28
136.96
135.92
131.75
129.42
128.59
127.99
122.12
119.20
117.57
77.35 CDCl3
77.03 CDCl3
76.71 CDCl3
56.72
44.42
f1 (ppm)

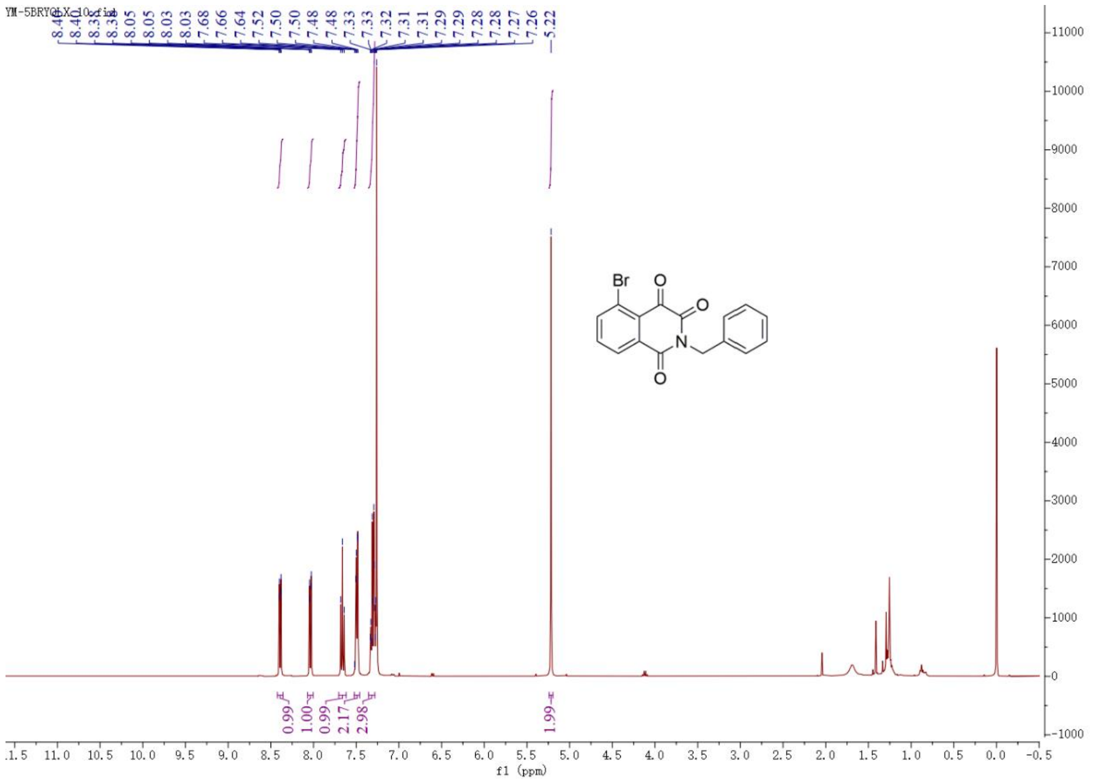
YM-5BRYQLX.10.fid
8.40
8.40
8.38
8.38
8.05
8.05
8.03
8.03
7.68
7.66
7.64
7.52
7.50
7.50
7.48
7.48
7.33
7.33
7.32
7.31
7.31
7.29
7.29
7.28
7.28
7.27
7.26
5.22
Br
O
O
N
O
0.99
1.00
0.99
2.17
2.98
1.99
f1 (ppm)

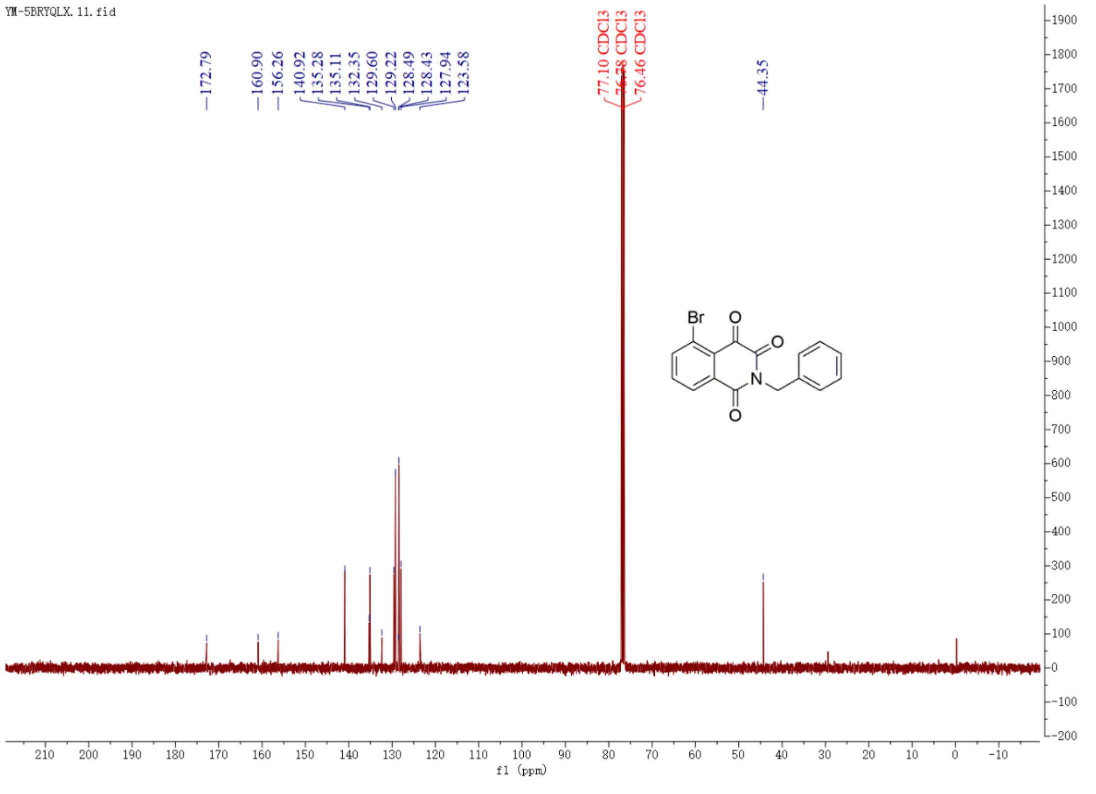
YM-5BRYQLX.11.fid
172.79
160.90
156.26
140.92
135.28
135.11
132.35
129.60
129.22
128.49
128.43
127.94
123.58
77.10 CDCl3
76.78 CDCl3
76.46 CDCl3
44.35
Br
O
O
N
O
f1 (ppm)

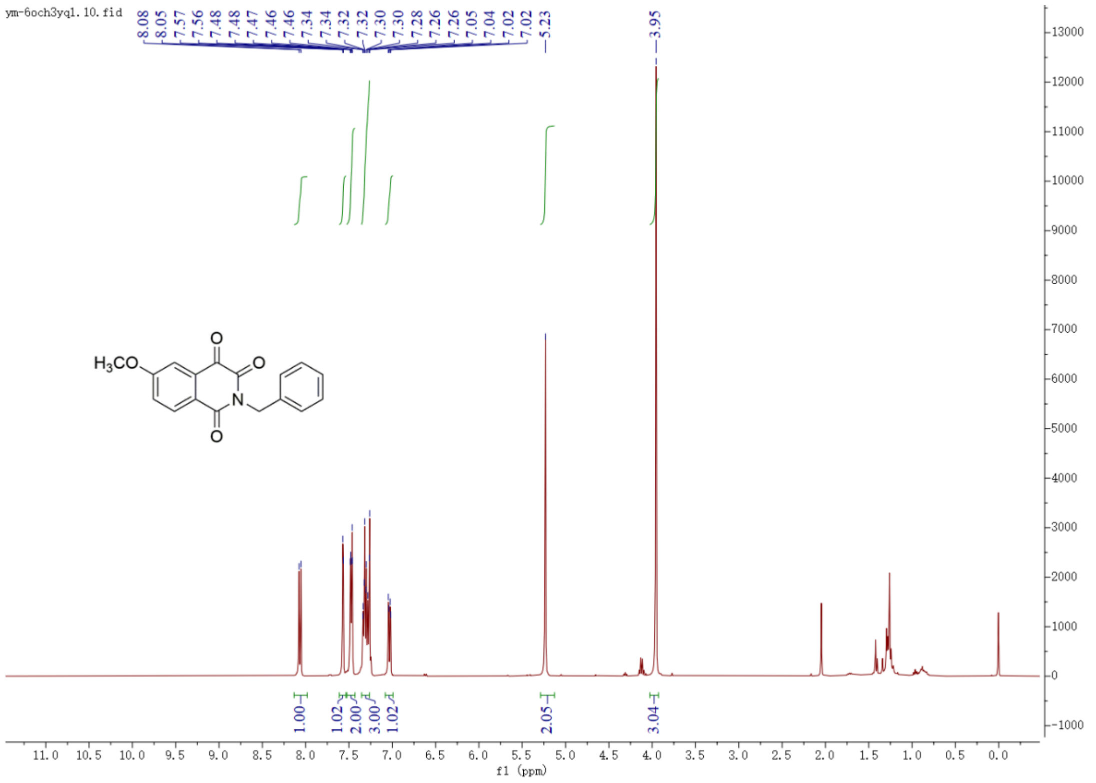
ym-6och3yql.10.fid
8.08
8.05
7.57
7.56
7.48
7.48
7.47
7.46
7.46
7.34
7.34
7.32
7.32
7.30
7.30
7.28
7.26
7.26
7.05
7.04
7.02
7.02
5.23
3.95
1.00
1.02
2.00
3.00
1.02
2.05
3.04
f1 (ppm)

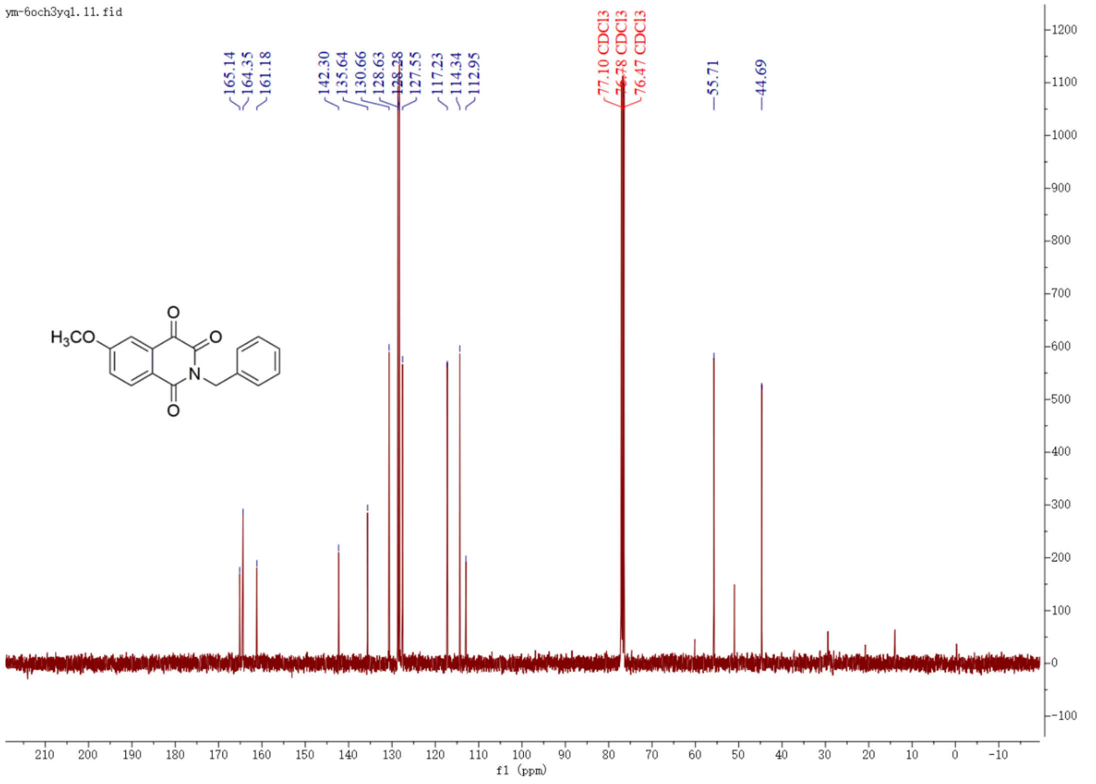
ym-6och3yql.11.fid
165.14
164.35
161.18
142.30
135.64
130.66
128.63
128.28
127.55
117.23
114.34
112.95
77.10 CDCl3
76.78 CDCl3
76.47 CDCl3
55.71
44.69
f1 (ppm)

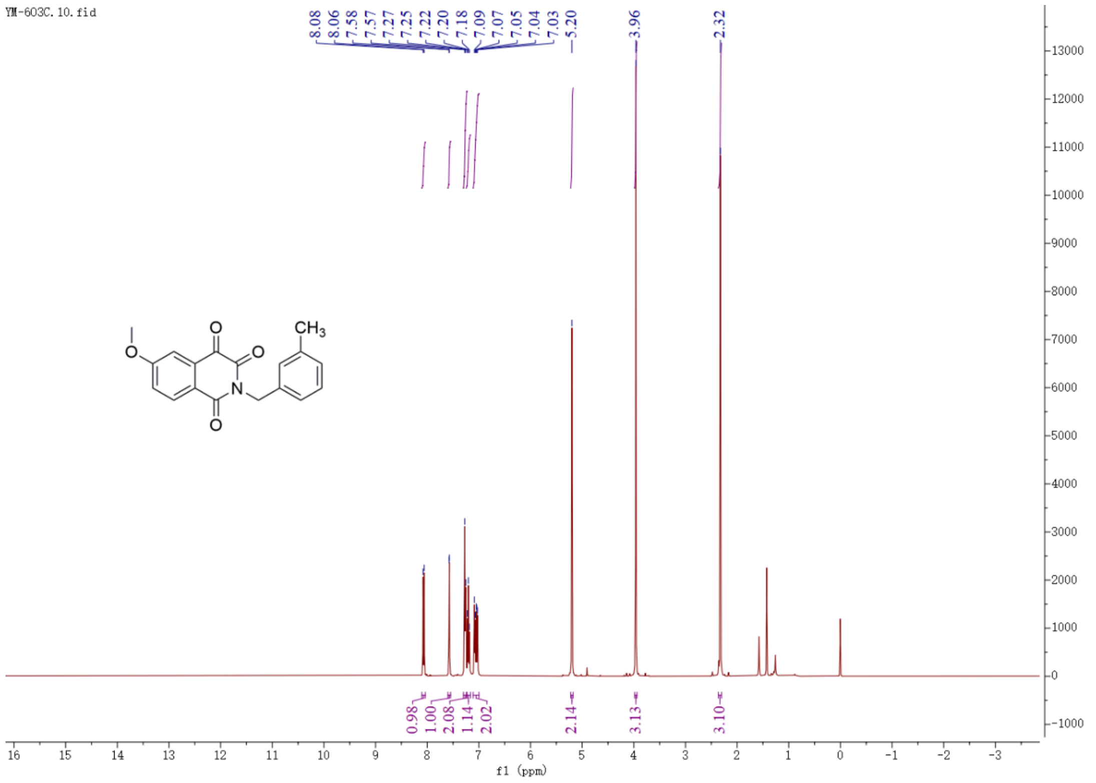
YM-603C.10.fid
8.08
8.06
7.58
7.57
7.27
7.25
7.22
7.20
7.18
7.09
7.07
7.05
7.04
7.03
5.20
3.96
2.32
CH3
O
N
0.98
1.00
2.08
1.14
2.02
2.14
3.13
3.10
f1 (ppm)

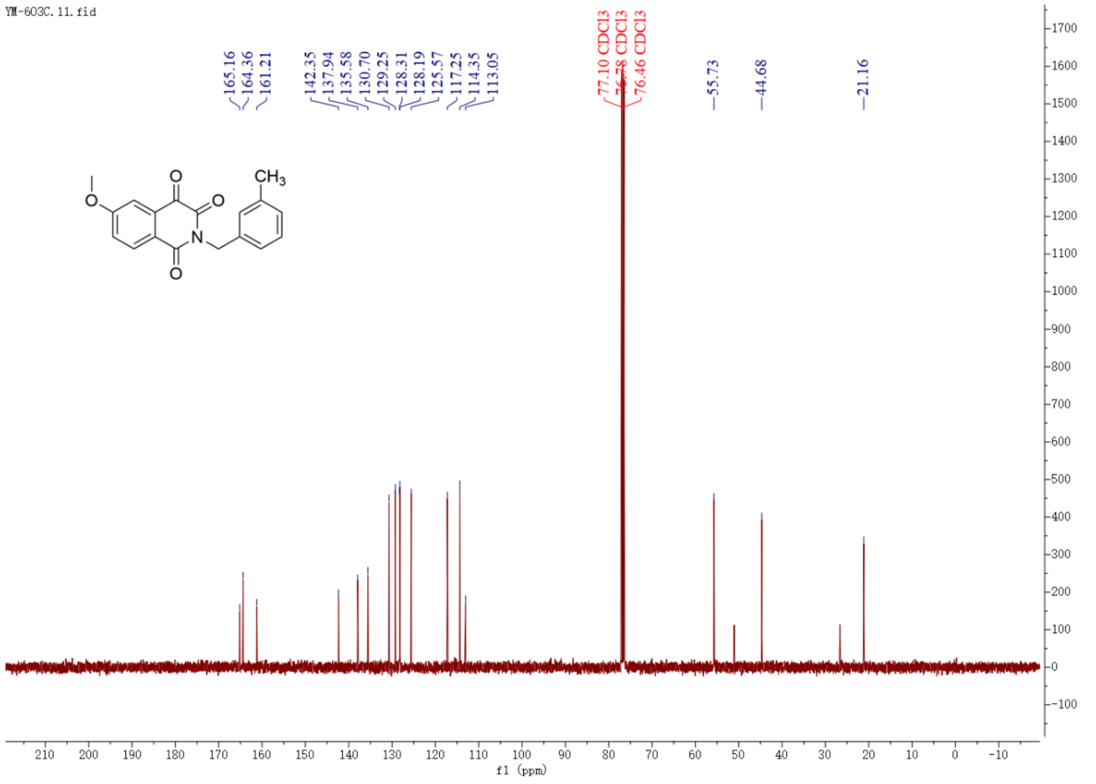
YM-603C.11.fid
165.16
164.36
161.21
142.35
137.94
135.58
130.70
129.25
128.31
128.19
125.57
117.25
114.35
113.05
77.10 CDCl3
76.78 CDCl3
76.46 CDCl3
55.73
44.68
21.16
CH3
O
N
f1 (ppm)

YM-603Br.10.fid

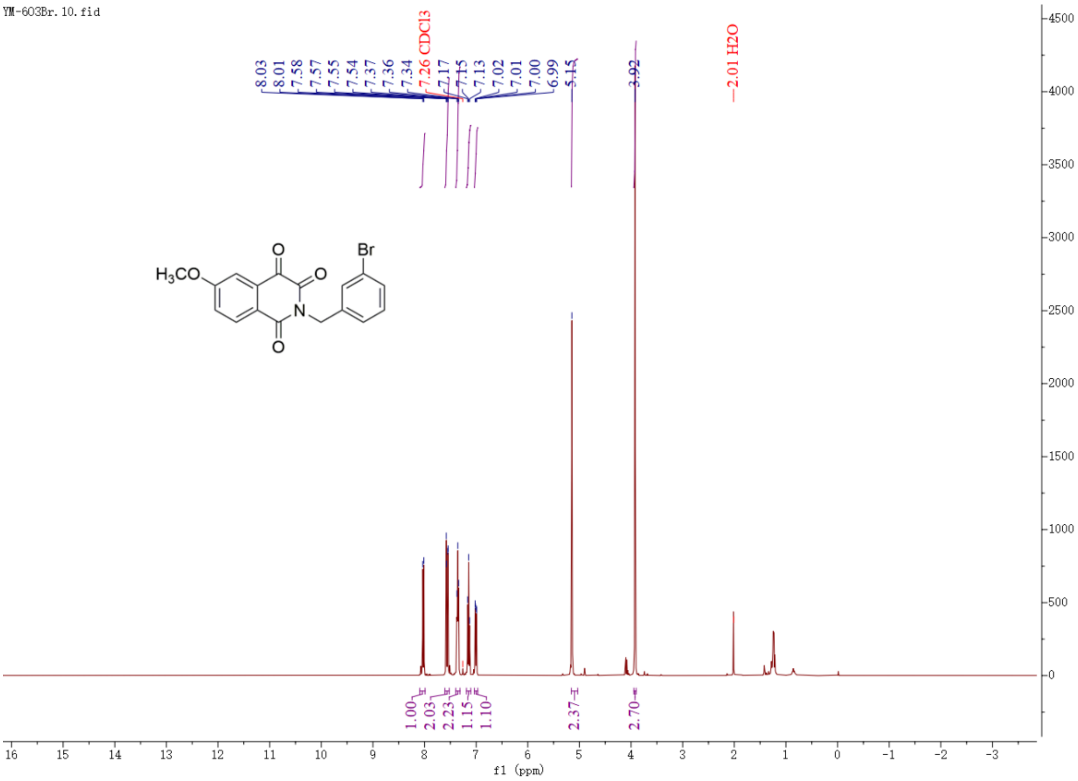

YM-603Br.11.fid

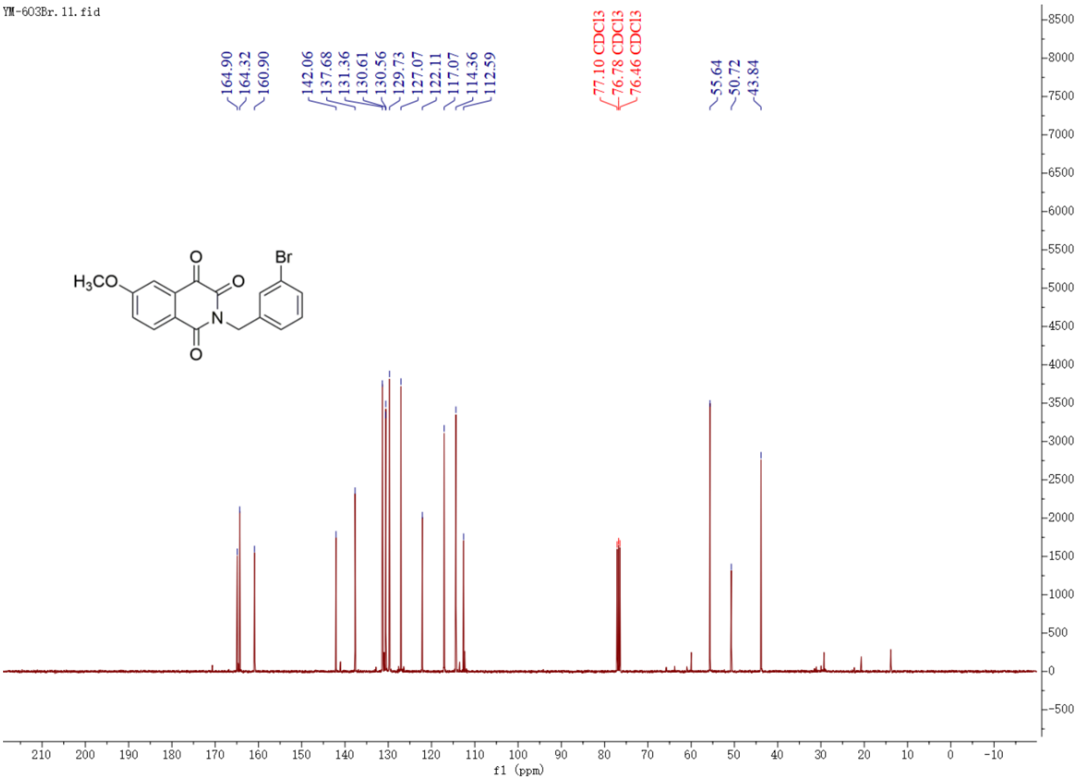

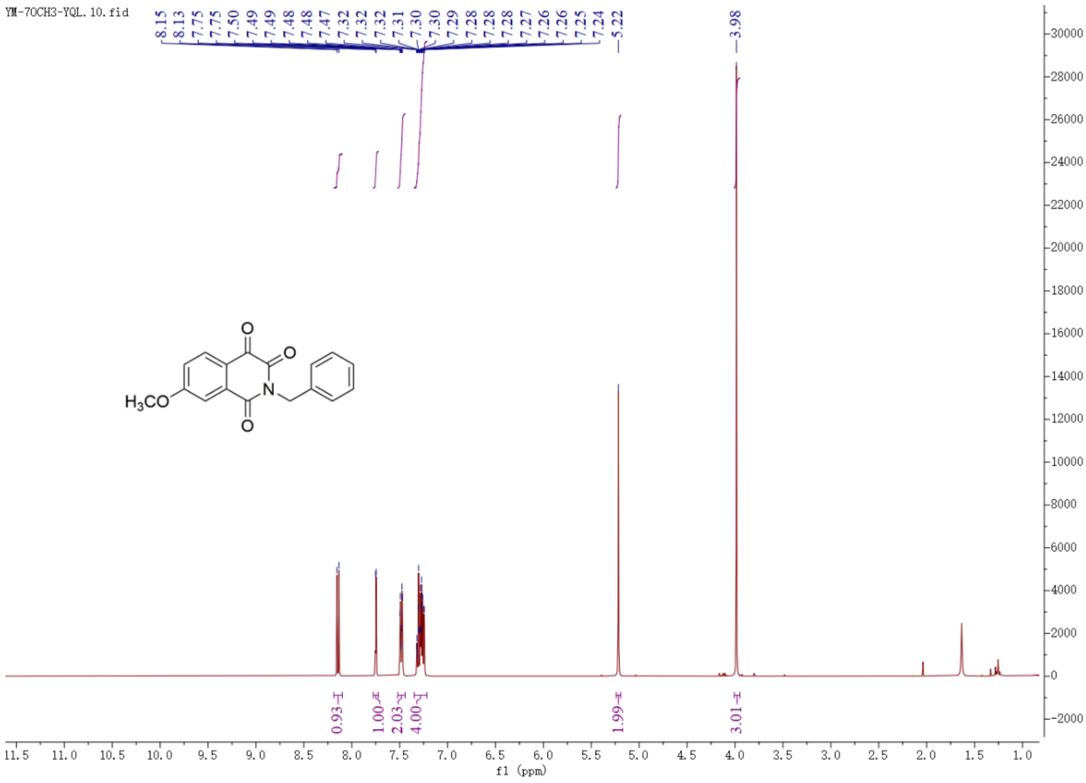
YM-7OCH3-YQL.10.fid
8.15
8.13
7.75
7.75
7.50
7.49
7.49
7.48
7.48
7.47
7.32
7.32
7.32
7.31
7.30
7.30
7.29
7.28
7.28
7.28
7.27
7.26
7.26
7.25
7.24
5.22
3.98
H3CO
O
O
O
N
0.93
1.00
2.03
4.00
1.99
3.01
f1 (ppm)

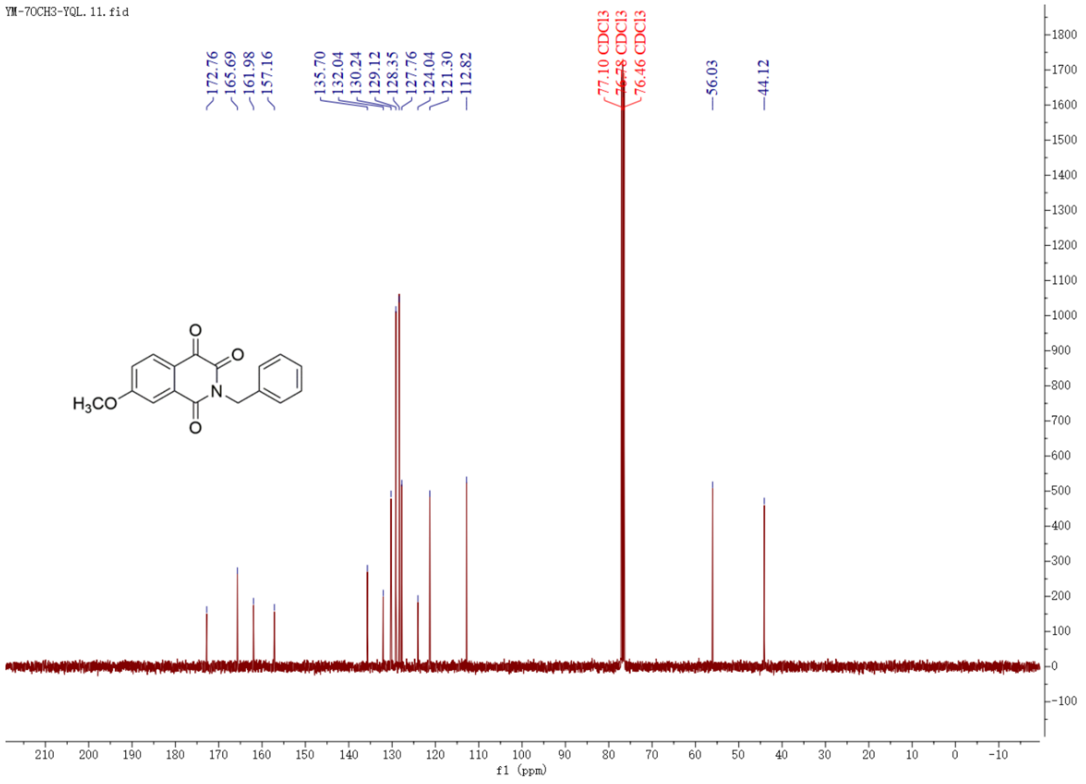
YM-7OCH3-YQL.11.fid
172.76
165.69
161.98
157.16
135.70
132.04
130.24
129.12
128.35
127.76
124.04
121.30
112.82
77.10 CDCl3
76.78 CDCl3
76.46 CDCl3
56.03
44.12
H3CO
O
O
O
N
f1 (ppm)

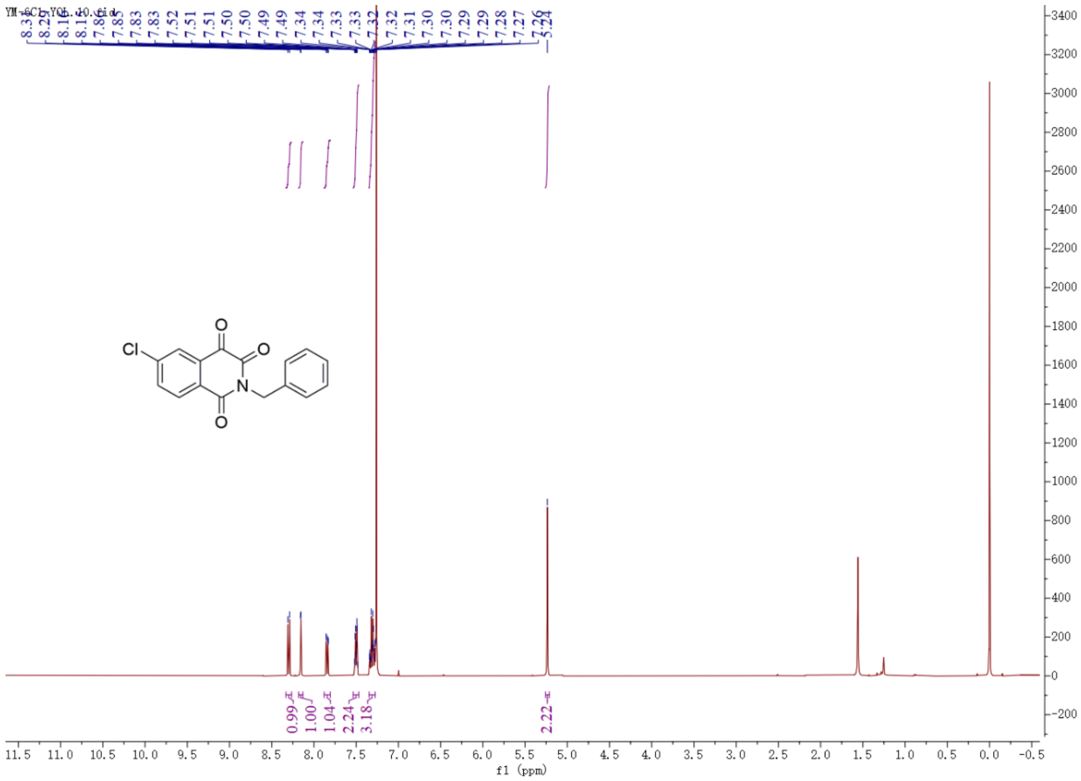
YM-6Cl-YQL.10.fid
8.31
8.29
8.16
8.15
7.85
7.85
7.83
7.83
7.52
7.51
7.51
7.50
7.50
7.49
7.34
7.34
7.33
7.33
7.32
7.32
7.31
7.30
7.30
7.29
7.29
7.28
7.27
7.26
5.24
Cl
O
N
0.99
1.00
1.04
2.24
3.18
2.22
f1 (ppm)

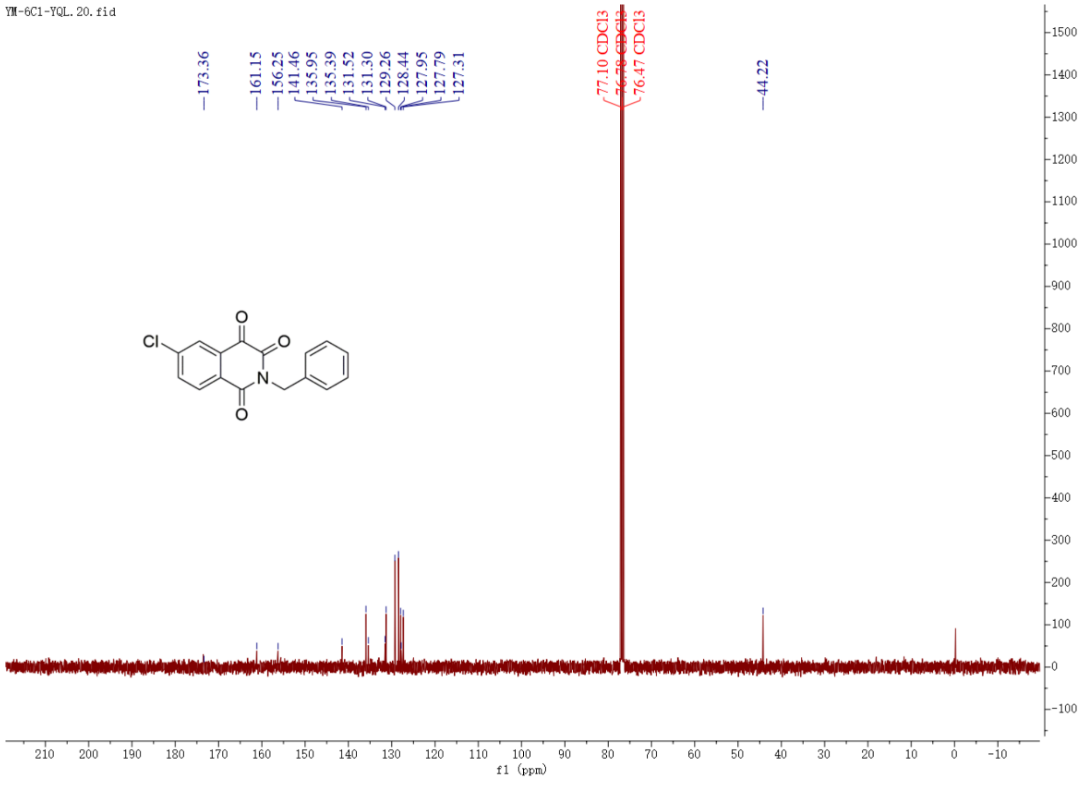
YM-6Cl-YQL.20.fid
173.36
161.15
156.25
141.46
135.95
135.39
131.52
131.30
129.26
128.44
127.95
127.79
127.31
77.10 CDCl3
76.78 CDCl3
76.47 CDCl3
44.22
Cl
O
N
f1 (ppm)

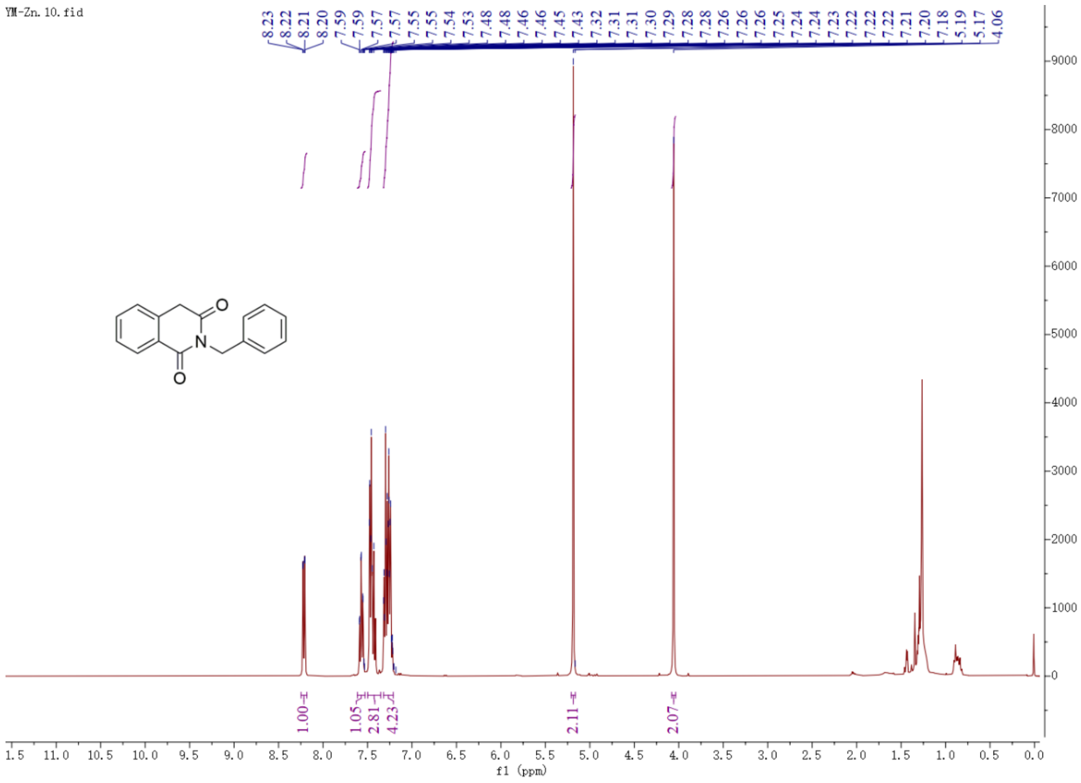

YM-Zn.10.fid
8.23
8.22
8.21
8.20
7.59
7.59
7.57
7.57
7.55
7.55
7.54
7.53
7.48
7.48
7.46
7.46
7.45
7.43
7.32
7.31
7.31
7.30
7.29
7.28
7.28
7.26
7.26
7.25
7.24
7.24
7.23
7.22
7.22
7.22
7.21
7.20
7.18
5.19
5.17
4.06
1.00
1.05
2.81
4.23
2.11
2.07
O
N
O
f1 (ppm)

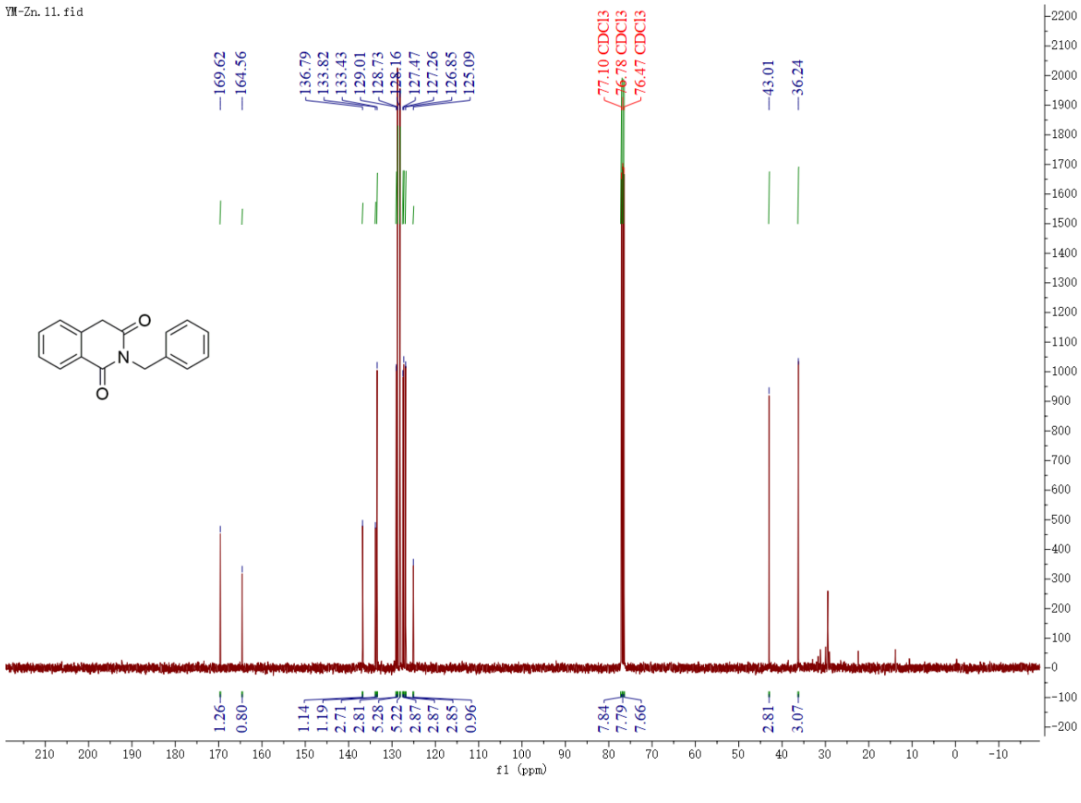

YM-Zn.11.fid
169.62
164.56
136.79
133.82
133.43
129.01
128.73
128.16
127.47
127.26
126.85
125.09
77.10 CDCl3
76.78 CDCl3
76.47 CDCl3
43.01
36.24
1.26
0.80
1.14
1.19
2.71
2.81
5.28
5.22
2.87
2.87
2.85
0.96
7.84
7.79
7.66
2.81
3.07
O
N
O
f1 (ppm)

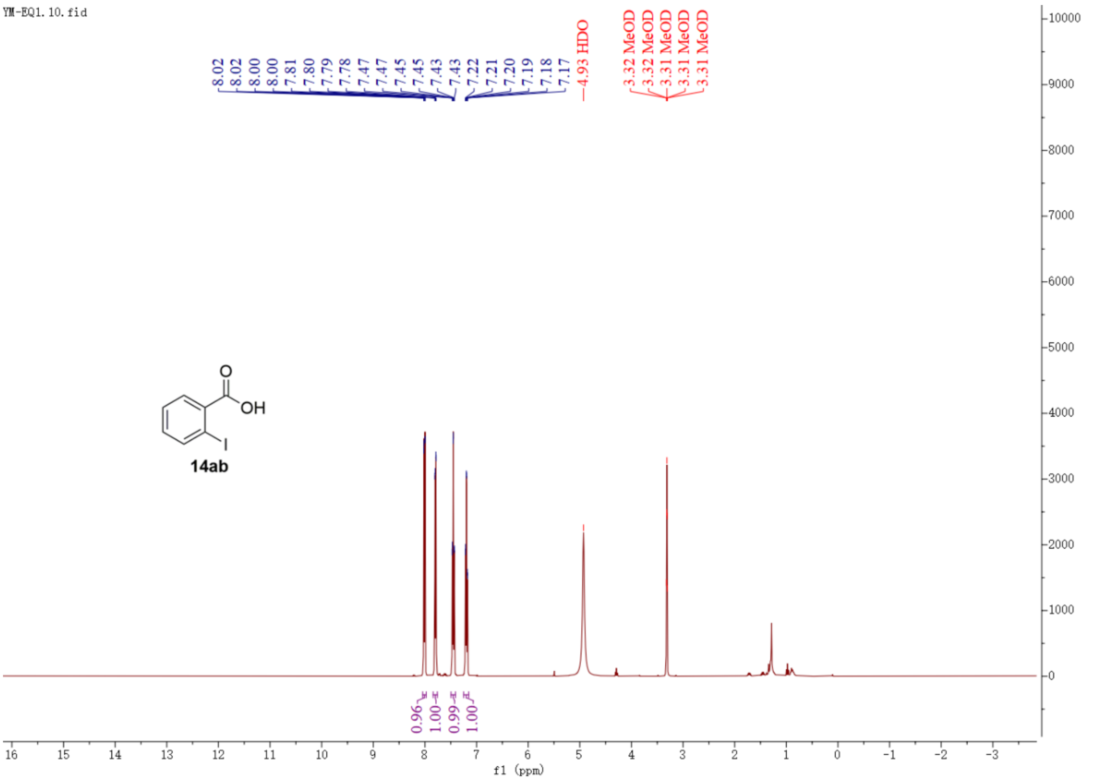
YM-EQ1.10.fid
8.02
8.02
8.00
8.00
7.81
7.80
7.79
7.78
7.47
7.47
7.45
7.45
7.43
7.43
7.22
7.21
7.20
7.19
7.18
7.17
4.93 HDO
3.32 MeOD
3.32 MeOD
3.31 MeOD
3.31 MeOD
3.31 MeOD
OH
O
I
14ab
0.96
1.00
0.99
1.00
f1 (ppm)

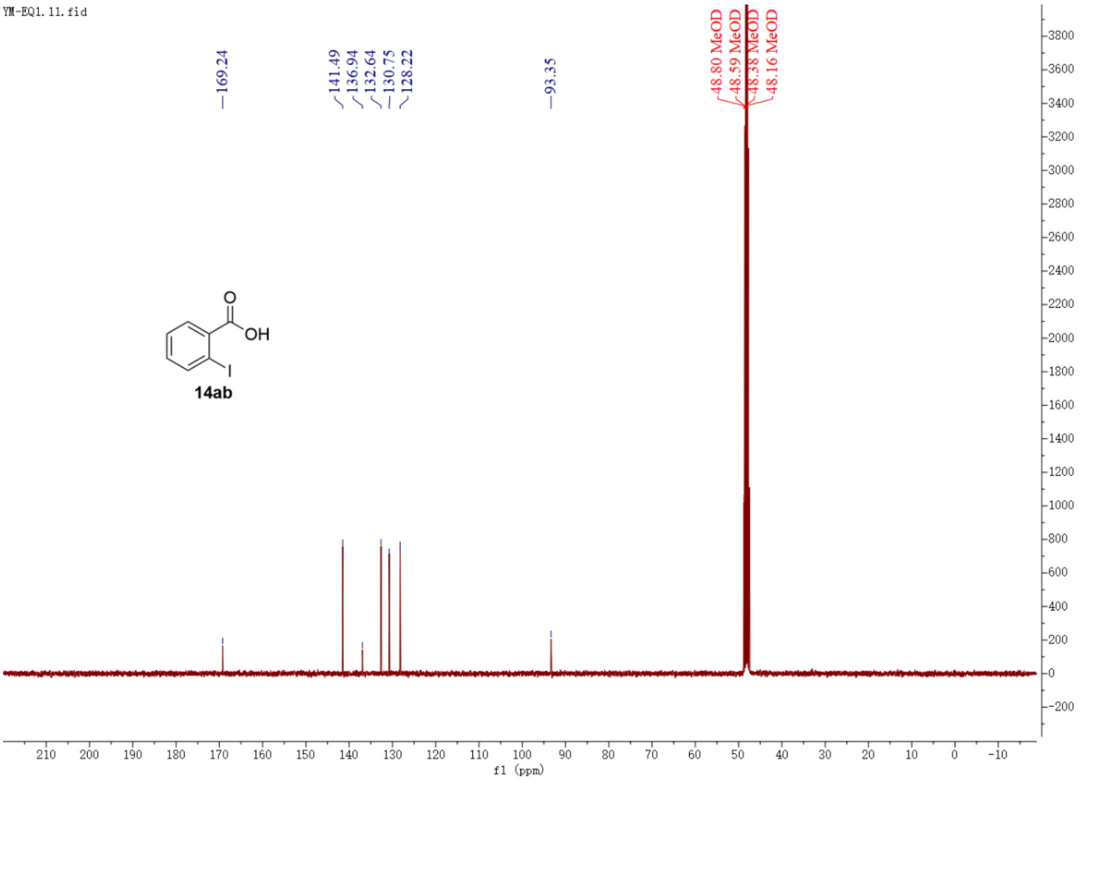
YM-EQ1.11.fid
169.24
141.49
136.94
132.64
130.75
128.22
93.35
48.80 MeOD
48.59 MeOD
48.38 MeOD
48.16 MeOD
OH
O
I
14ab
f1 (ppm)

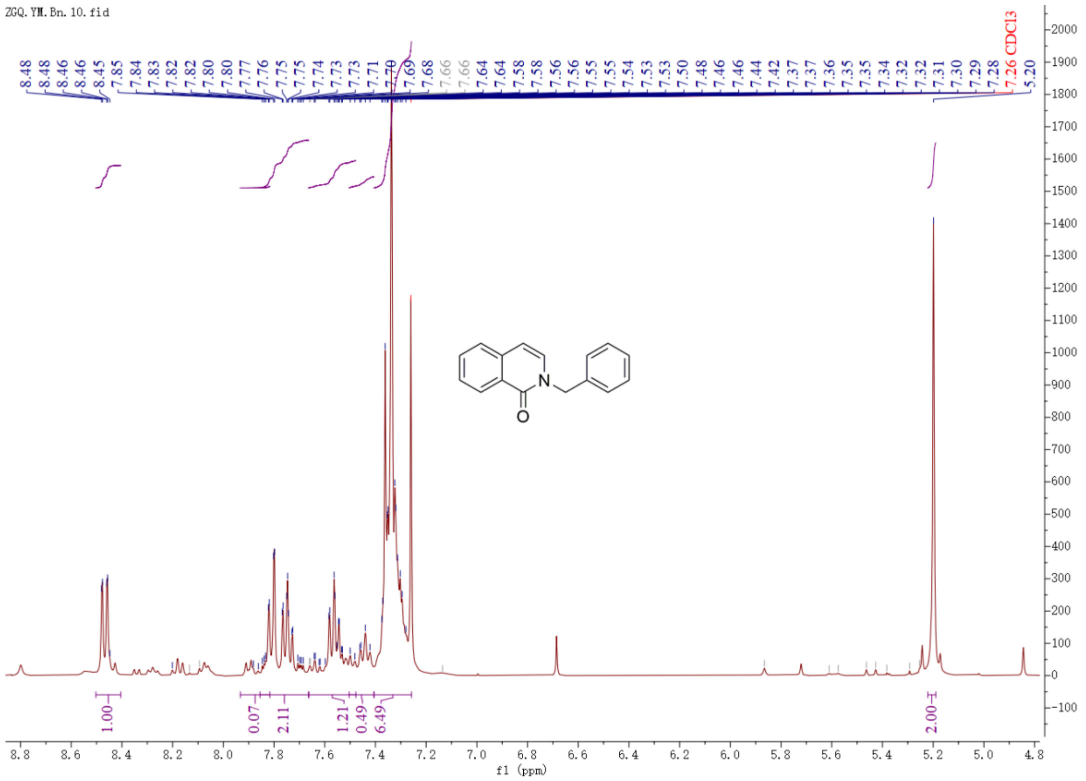
ZGQ.YM.Bn.10.fid
8.48
8.48
8.46
8.46
8.45
7.85
7.84
7.83
7.82
7.82
7.80
7.77
7.76
7.75
7.75
7.74
7.73
7.73
7.71
7.70
7.69
7.68
7.66
7.66
7.64
7.64
7.58
7.58
7.56
7.56
7.55
7.55
7.54
7.53
7.53
7.50
7.48
7.46
7.44
7.42
7.37
7.37
7.36
7.35
7.35
7.34
7.32
7.32
7.31
7.30
7.29
7.28
7.26 CDCl3
5.20
1.00
0.07
2.11
1.21
0.49
6.49
2.00
f1 (ppm)

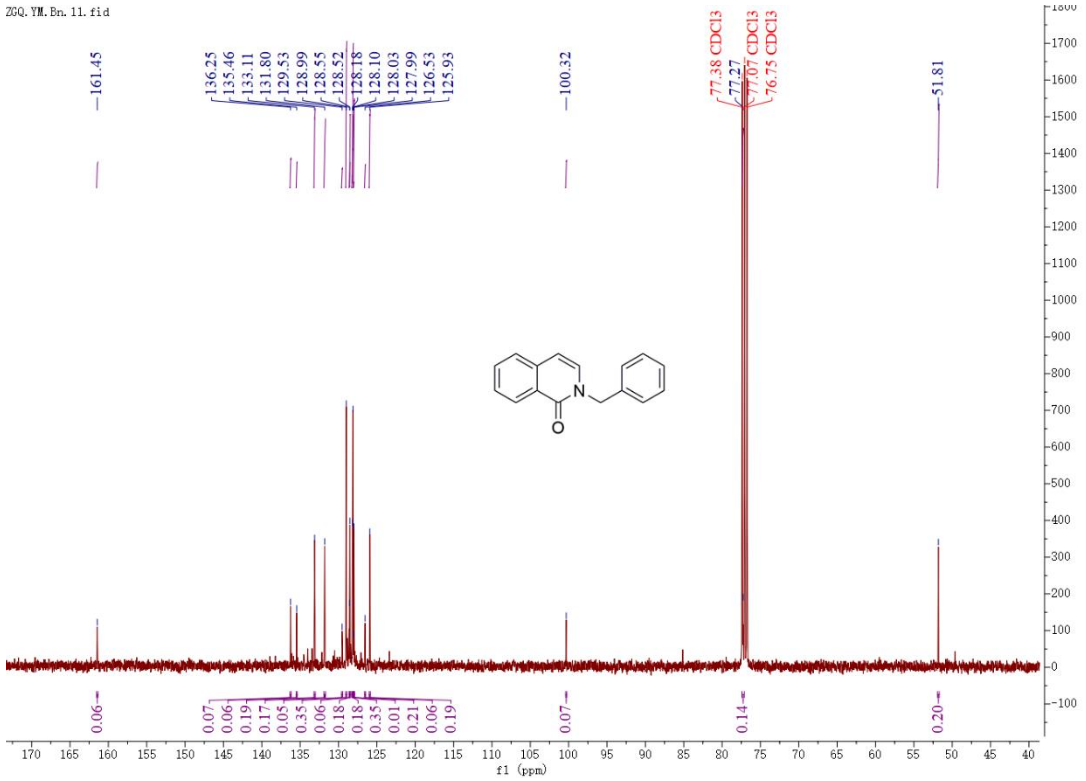
ZGQ.YM.Bn.11.fid
161.45
136.25
135.46
133.11
131.80
129.53
128.99
128.55
128.52
128.18
128.10
128.03
127.99
126.53
125.93
100.32
77.38 CDCl3
77.27
77.07 CDCl3
76.75 CDCl3
51.81
0.06
0.07
0.06
0.19
0.17
0.05
0.35
0.06
0.18
0.18
0.35
0.01
0.21
0.06
0.19
0.07
0.14
0.20
f1 (ppm)

2. Single crystal structure and data

Single crystal structure of compound 3aa

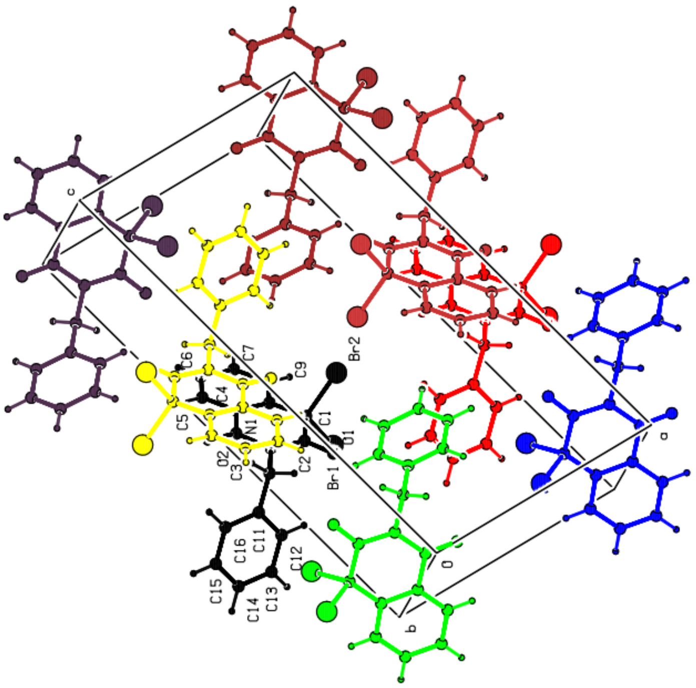

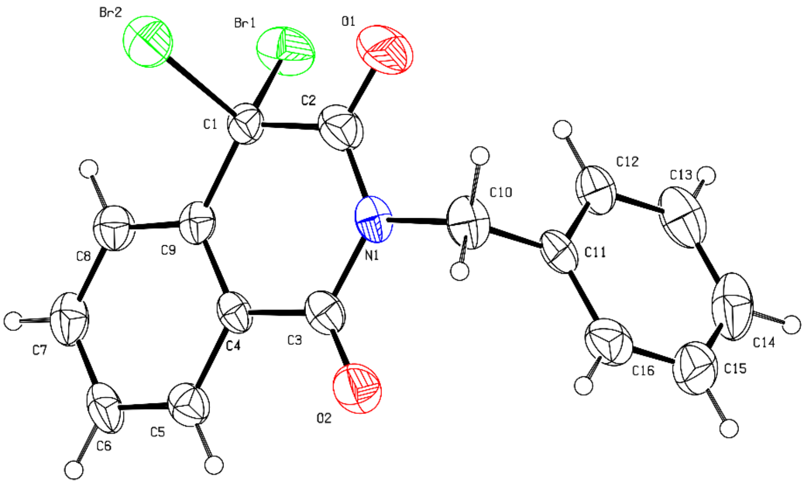

Compound 3aa single crystal data

Identification code	211027_s2_ycm
Empirical formula	C16H11Br2NO2
Formula weight	409.08
Temperature/K	293.15
Crystal system	monoclinic
Space group	P21/c
a/Å	9.1422（10）
b/Å	9.3844（9）
c/Å	17.8359（18）
α/°	90
β/°	102.960（10）
γ/°	90
Volume/Å3	1491.2（3）
Z	4
ρcalcg/cm3	1.822
μ/mm-1	5.439
F（000）	800.0
Crystal size/mm3	0.35 × 0.3 × 0.25
Radiation	MoKα （λ = 0.71073）
2Θ range for data collection/°	6.306 to 52.744
Index ranges	-11 ≤ h ≤ 11, -10 ≤ k ≤ 11, -22 ≤ l ≤ 21
Reflections collected	6451
Independent reflections	3054 [Rint = 0.0481, Rsigma = 0.1031]
Data/restraints/parameters	3054/0/190
Goodness-of-fit on F2	0.967
Final R indexes [I>=2σ （I）]	R1 = 0.0553, wR2 = 0.0966
Final R indexes [all data]	R1 = 0.1401, wR2 = 0.1241
Largest diff. peak/hole / e Å-3	0.41/-0.58
